# Photons, Excitons, and Electrons in Covalent Organic Frameworks

**DOI:** 10.1021/jacs.3c14833

**Published:** 2024-11-18

**Authors:** Dominic Blätte, Frank Ortmann, Thomas Bein

**Affiliations:** † Department of Chemistry and Center for NanoScience, University of Munich (LMU), Butenandtstr. 5-13, 81377 Munich, Germany; ‡ Department of Chemistry, TUM School of Natural Sciences, Technical University of Munich, Lichtenbergstr. 4, 85748 Garching, Germany

## Abstract

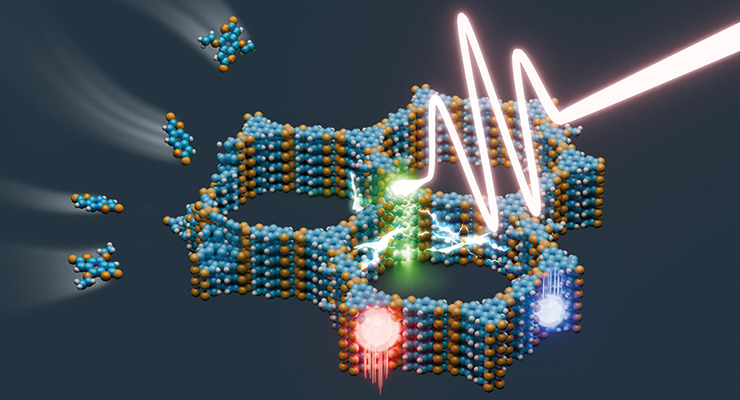

Covalent organic frameworks (COFs) are created by the condensation of molecular building blocks and nodes to form two-dimensional (2D) or three-dimensional (3D) crystalline frameworks. The diversity of molecular building blocks with different properties and functionalities and the large number of possible framework topologies open a vast space of possible well-defined porous architectures. Besides more classical applications of porous materials such as molecular absorption, separation, and catalytic conversions, interest in the optoelectronic properties of COFs has recently increased considerably. The electronic properties of both the molecular building blocks and their linkage chemistry can be controlled to tune photon absorption and emission, to create excitons and charge carriers, and to use these charge carriers in different applications such as photocatalysis, luminescence, chemical sensing, and photovoltaics. In this Perspective, we will discuss the relationship between the structural features of COFs and their optoelectronic properties, starting with the building blocks and their chemical connectivity, layer stacking in 2D COFs, control over defects and morphology including thin film synthesis, exploring the theoretical modeling of structural, electronic, and dynamic features of COFs, and discussing recent intriguing applications with a focus on photocatalysis and photoelectrochemistry. We conclude with some remarks about present challenges and future prospects of this powerful architectural paradigm.

## Introduction

Organic semiconductors are extremely versatile and sustainable materials with many intriguing properties.^1^ They have been used in organic photovoltaics, organic photodetectors, organic electronics, organic light emitting diodes and many other applications, including energy storage and photocatalysis. However, their structures, morphologies and properties are difficult to control because they are often not highly crystalline, and typically contain significant fractions of amorphous phases.

In contrast, crystalline covalent organic frameworks (COFs) are characterized by atomistic definition of the positioning of every molecular moiety in the structure, at least in principle. These materials have been discovered by the group of Yaghi at around 2005^2^ and have since seen an enormous surge in scientific interest.^3−6^ They are created by the condensation of molecular building blocks that form crystalline frameworks with well-defined pore sizes, pore topologies and different unit cell dimensions.

**Scheme 1:**
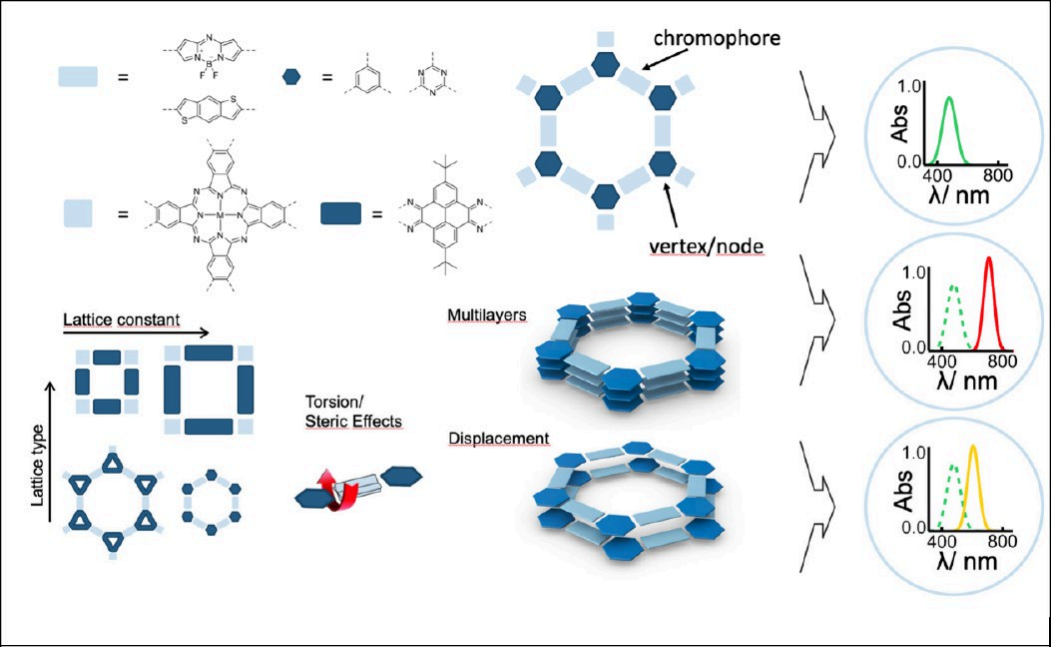
Some of the Key Structural Features Controlling the Optoelectronic Properties of 2D COFs Key structural features include linear and multidentate building blocks (vertices), lattice topology, lattice size and geometry, steric features such as torsion angles impacting electronic coupling, light-harvesting properties of building blocks (chromophores), and the impact of layer π-stacking mode on non-covalent electronic coupling. Often these effects can be directly observed in the optical absorption spectra, schematically shown at the right.

In this perspective, we will explore the relationship between the molecular structure of covalent organic frameworks and their optoelectronic properties.^7^ In the beginning we will focus on ways to achieve structural control over these materials, ranging from their building blocks and linkage chemistry to pore topology, defect density and morphology, and their impact on specific optoelectronic properties. To set the stage, some of the key structural features controlling the physical properties of COFs are shown in Scheme 1. A detailed theoretical understanding of electronic structure, excited state properties and charge carrier mobilities and their relationship to framework topology is key to achieving predictive control over optoelectronic properties and functionality such as light harvesting, luminescence, chemical sensing, or light-induced charge carrier extraction in photovoltaics and photocatalysis. The latter properties will be discussed in subsequent sections.

## Achieving Structural Control of Optoelectronic Properties: Synthesis

### Two-Dimensional Covalent Organic Frameworks in Bulk Form

**Figure 1:**
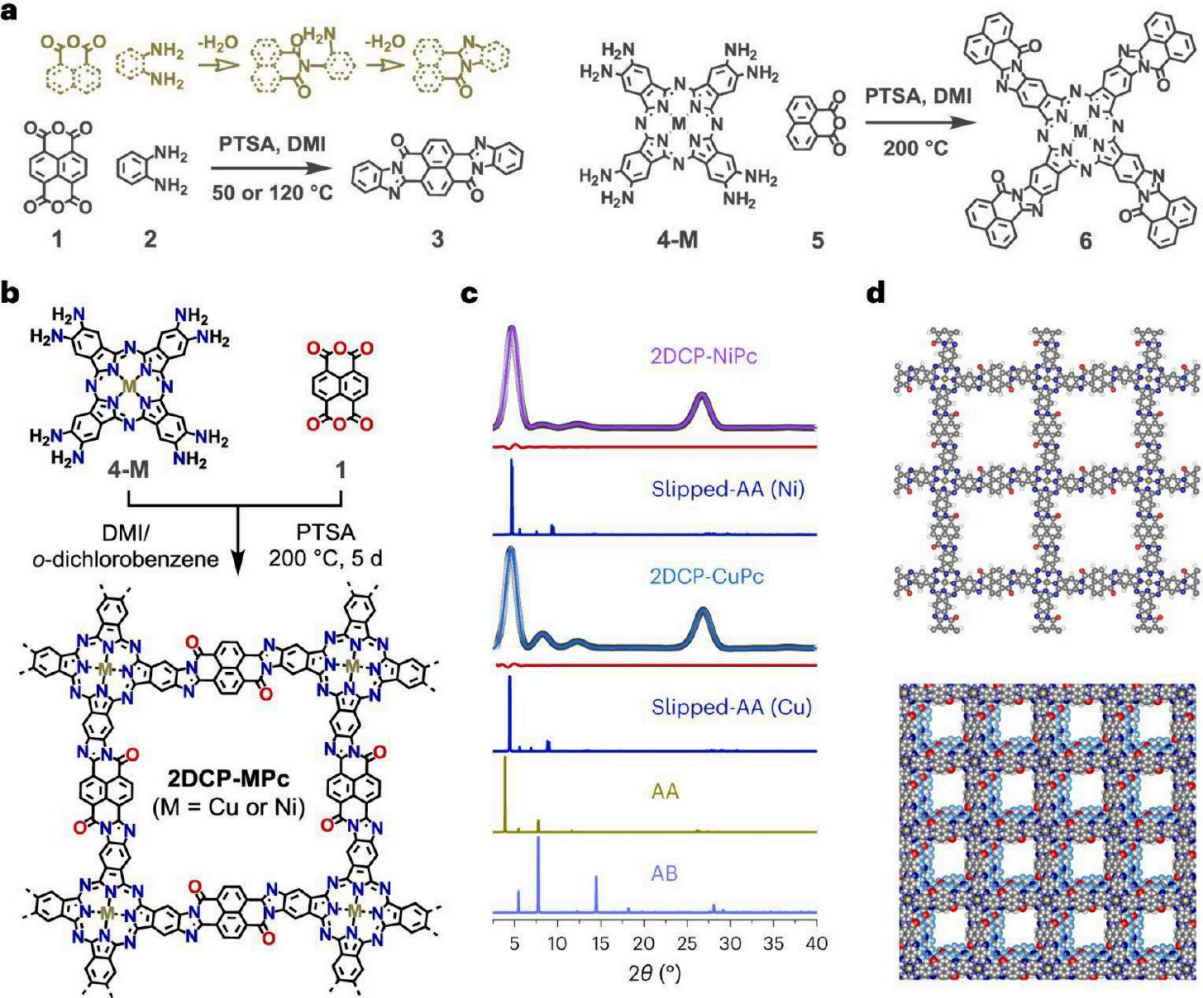
Phthalocyanine-based poly(benzimidazobenzophenanthroline)-ladder-type two-dimensional conjugated polymers (with M = Cu, Ni), constructed from octaamino-phthalocyaninato metal(II) and naphthalenetetracarboxylic dianhydride by polycondensation under solvothermal conditions. (a, b) Schematics of synthesis. (c) Experimental (violet and blue lines), Pawley refined (black dotted lines), and calculated (dark blue lines for slipped AA stacking as well as for AA and AB stacking) PXRD patterns as well as their difference plots (dark red lines) of the COFs. (d) Models of the monolayer and slipped-AA-stacked COFs. Reproduced from ref (18). CC BY 4.0.

The design of ***building blocks and nodes*** has a profound effect on the structure and properties of COFs. Rather simple building blocks such as benzene, triphenylene, triphenyl benzene or tetraphenyl pyrene have been some of the first building blocks for the synthesis of COFs, while later work has embraced the use of tetrafunctional porphyrin and metal-containing porphyrin nodes^8^ as well as phthalocyanine nodes.^9^ Building blocks such as diketopyrrolopyrrole,^10^ Janus diones,^11^ antiaromatic dibenzopentalenes,^12^ polyphenylenes,^13^ benzotrithiophene,^14^ switchable spiropyran,^15^ thiophene backbone,^16^ N-confused metalloporphyrins,^17^ ladder-type moieties^18^ (Figure 1), star-shaped acceptor moieties^19^ and double-walled structures,^20^ isoindigo and its variants,^21^ as well as diverse intraplane donor–acceptor combinations^22^ have been used to construct two-dimensional COFs. Intriguing helical COFs based on novel construction paradigms have recently been reported.^23^ The ***linkage chemistry***(24) has evolved from the formation of boronic acid esters by combining vicinal diols with boronic acids (or formation of boroxine rings)^2,25^—recently further developed using transesterification^26^—to the formation of imines via the condensation of primary amines and aldehydes.^27^

**Figure 2:**
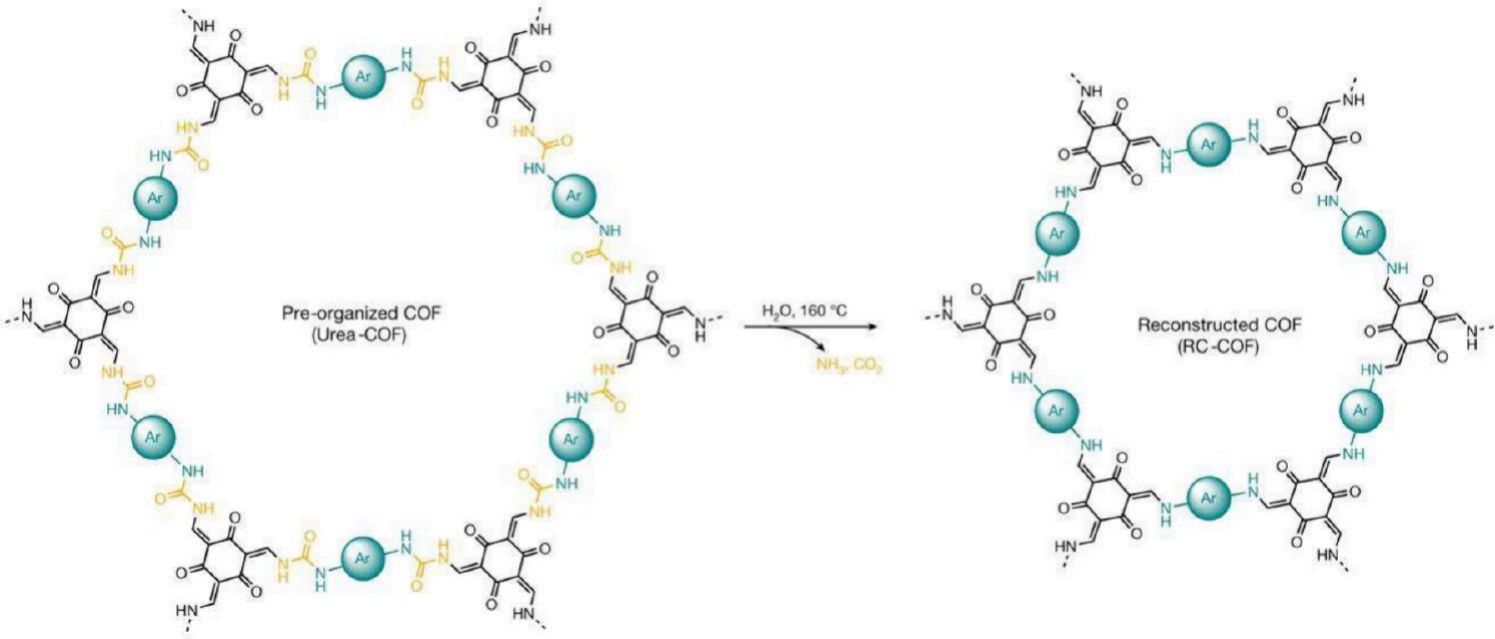
Chemical reconstruction of COFs. Here, monomers are preorganized using reversible urea linkages to form a highly crystalline framework, followed by solvothermal treatment to remove the urea tethers (producing NH_3_ and CO_2_; Ar = linear aromatic moieties), thereby releasing monomers that undergo *in situ* polymerization to form the reconstructed β-ketoenamine COF. Adapted from ref (54). CC BY 4.0.

A key feature of this linkage chemistry is the dynamic nature of the bond formation (or slightly reversible bond formation), which is important for the generation of crystalline, two-dimensional layers and allows for repair or error correction during the synthesis^28^ of COFs. However, this reversible nature of the linkage bonds often also implies limited stability toward the reverse reaction, i.e., decomposition. To address this issue, a number of subsequent “locking-in” reactions have been developed, which allow for the formation of more stable bonds in the COF lattice, including linkages based on the moieties shown in Table 1 below (along with sequential reactions containing reversible steps). Additional linkages include iminium,^29^ nitrone,^30−32^ benzobisthiazole (sp^2^-carbon conjugated),^33^ aminal,^34^ benzobisoxazole-vinylene,^35^ dioxin,^36^ ester,^37^ porphyrazine,^38^ aza-bridged bis(phenanthroline) macrocycle-linked,^39^ cyanurate moieties,^40^ ketazine,^41^ as well as those generated via multicomponent^42^ or light-induced multicomponent reactions.^43^ Regarding optoelectronic properties, the degree of conjugation between building blocks, as defined by the COF-linkage, is of key importance. A very important development in this regard was the formation of C=C double bonds achieved through Knoevenagel-type condensation reactions between C–H acidic moieties and aldehydes.^44,45^ The discovery of these reactions opened up the design of COFs to fully C=C conjugated systems, and has been complemented with emerging alternative strategies.^46−48^ Vinylene-linked COFs have also been obtained via tetrazine linkers,^49^ and Wittig-reactions combining aldehydes and ylides.^50^ C=C bond-linked COFs can be reduced to C–C bond linked COFs with NaBH_4_ in a crystal-to-crystal transformation.^51^ The direct formation of stable C–C single-bonded COFs was recently achieved, employing methyl viologen dichloride with acidic methyl groups (due to direct connection to N^+^ ionic sites) as linear linker for aldehyde-bearing nodes.^52^ Three-component concerted reactions have been developed for the construction of pyrene-fused azaacene COFs.^53^ Finally, an original avenue toward building imine-based COFs via reconstructing precursor frameworks containing a removable covalent tether was reported (Figure 2).^54^ Here, a chemical reconstruction in preorganized urea-linked COFs upon solvothermal treatment initiates a multistep urea hydrolysis reaction followed by imine condensation that produces highly crystalline COFs.

**Table 1:**
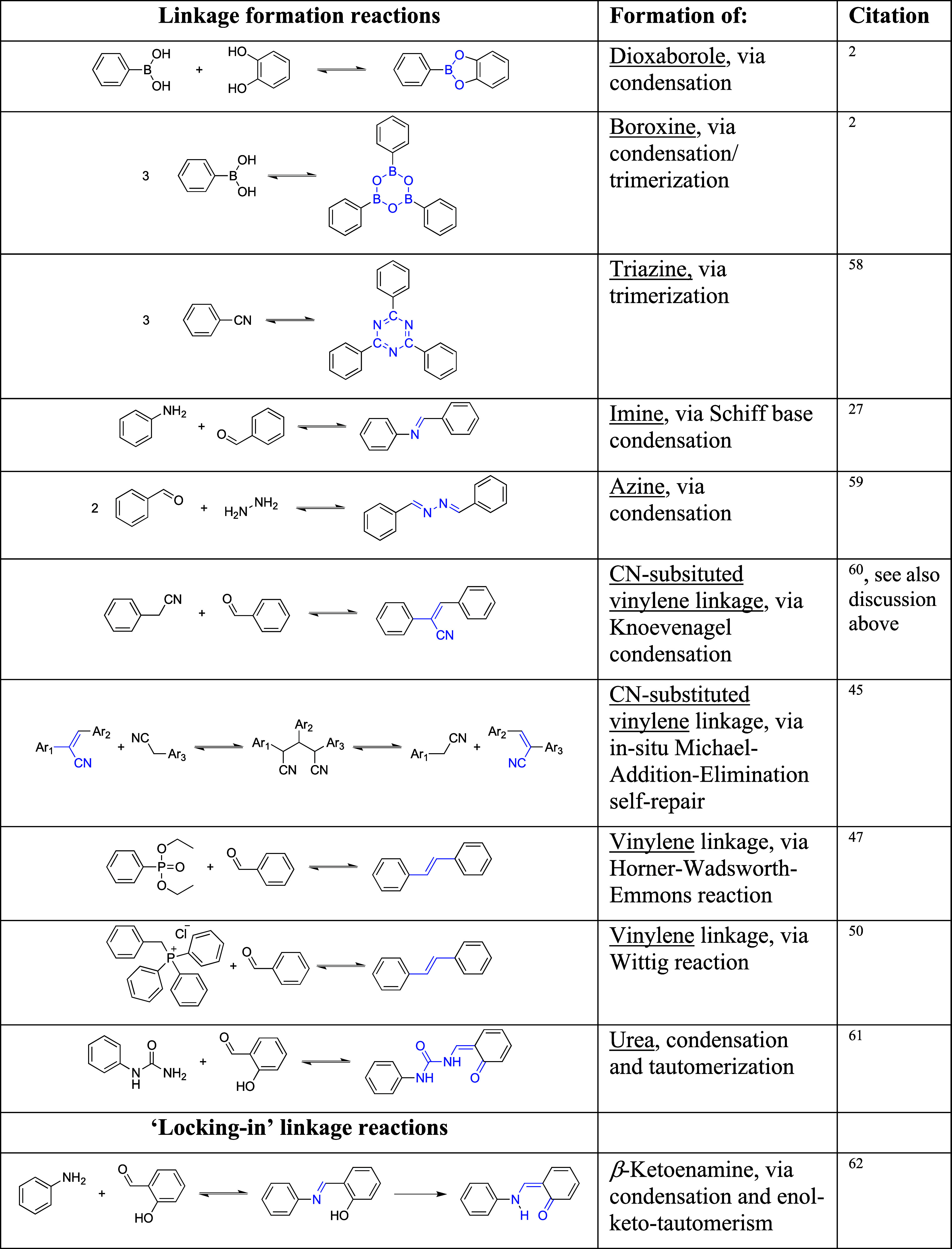

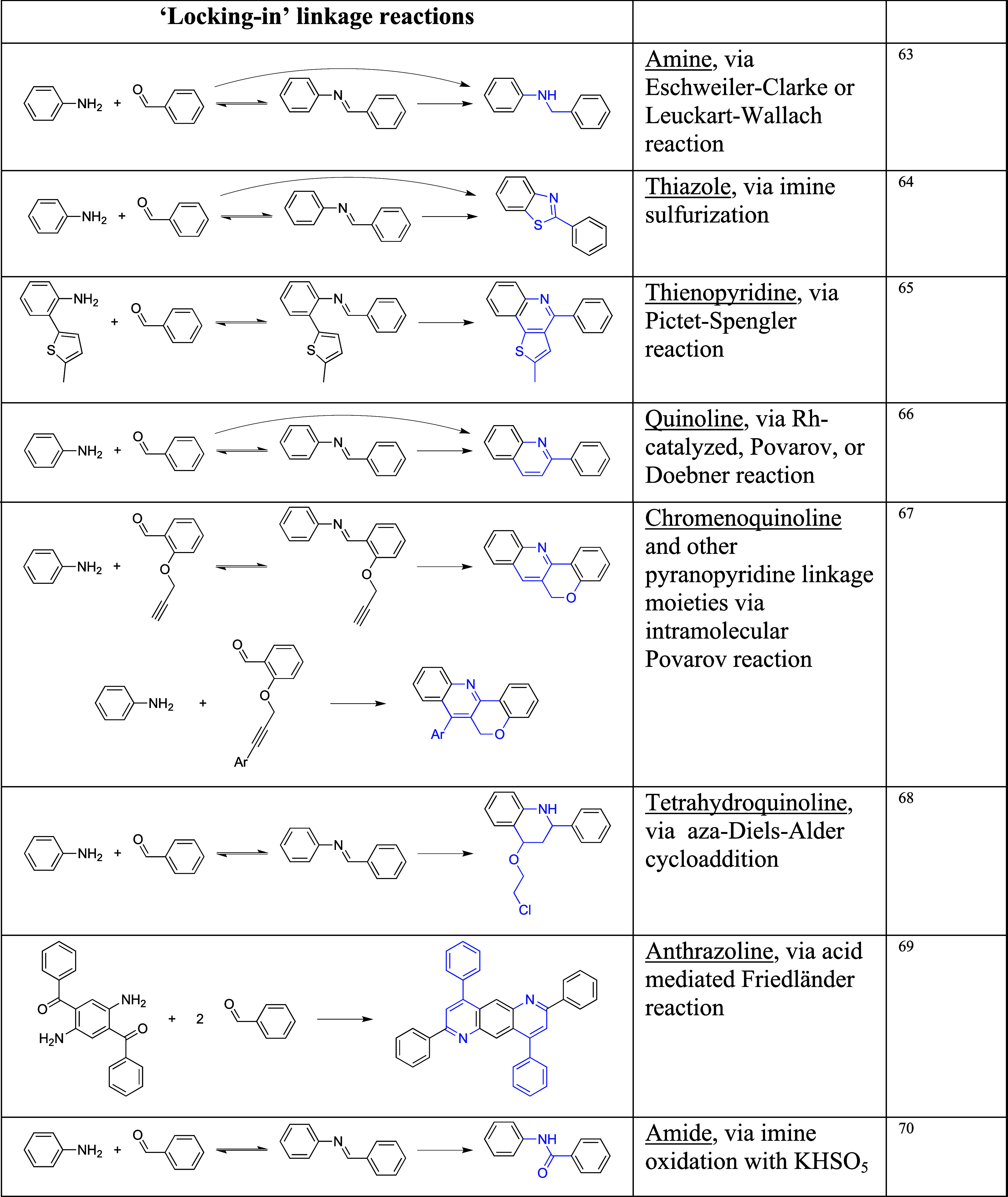

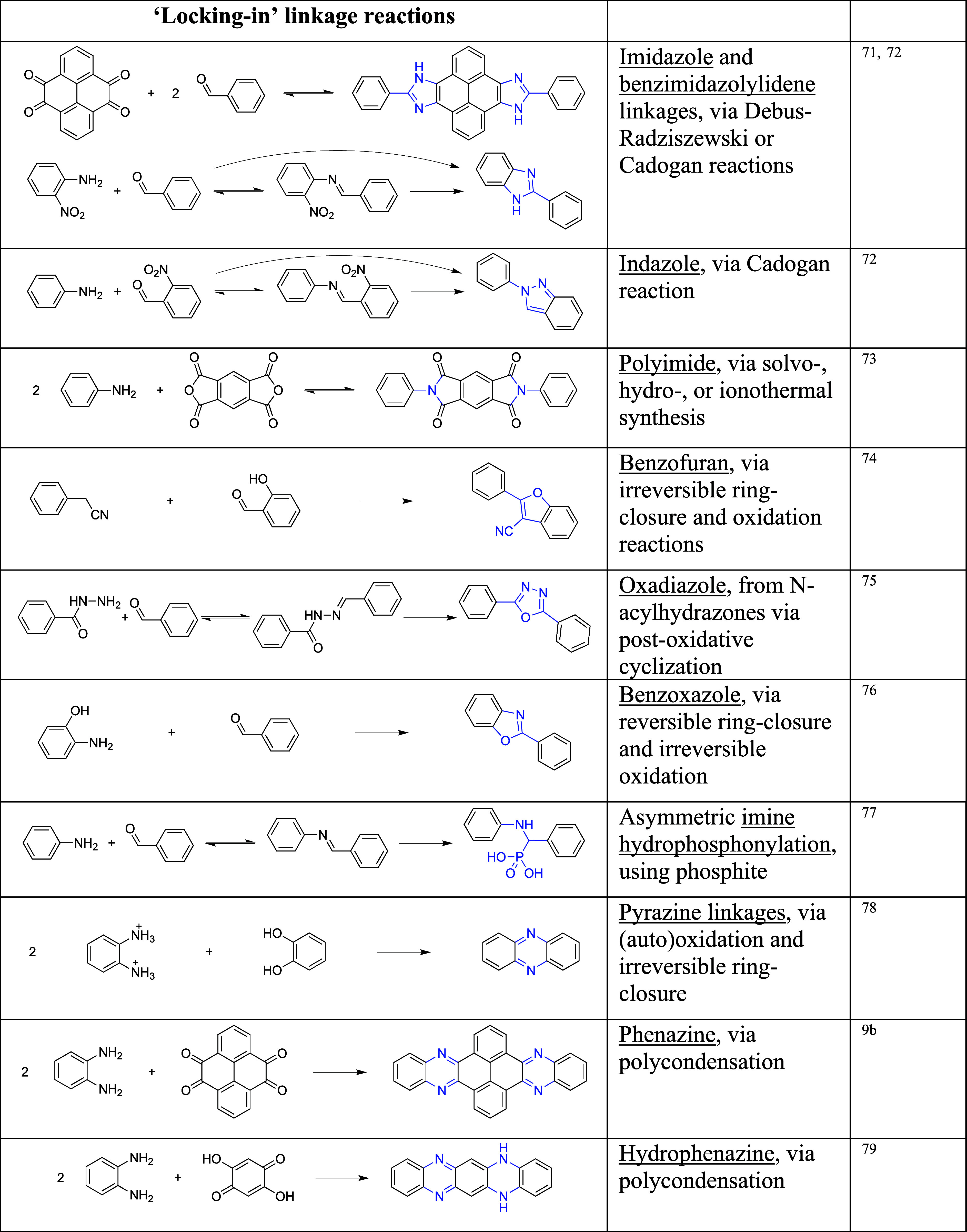

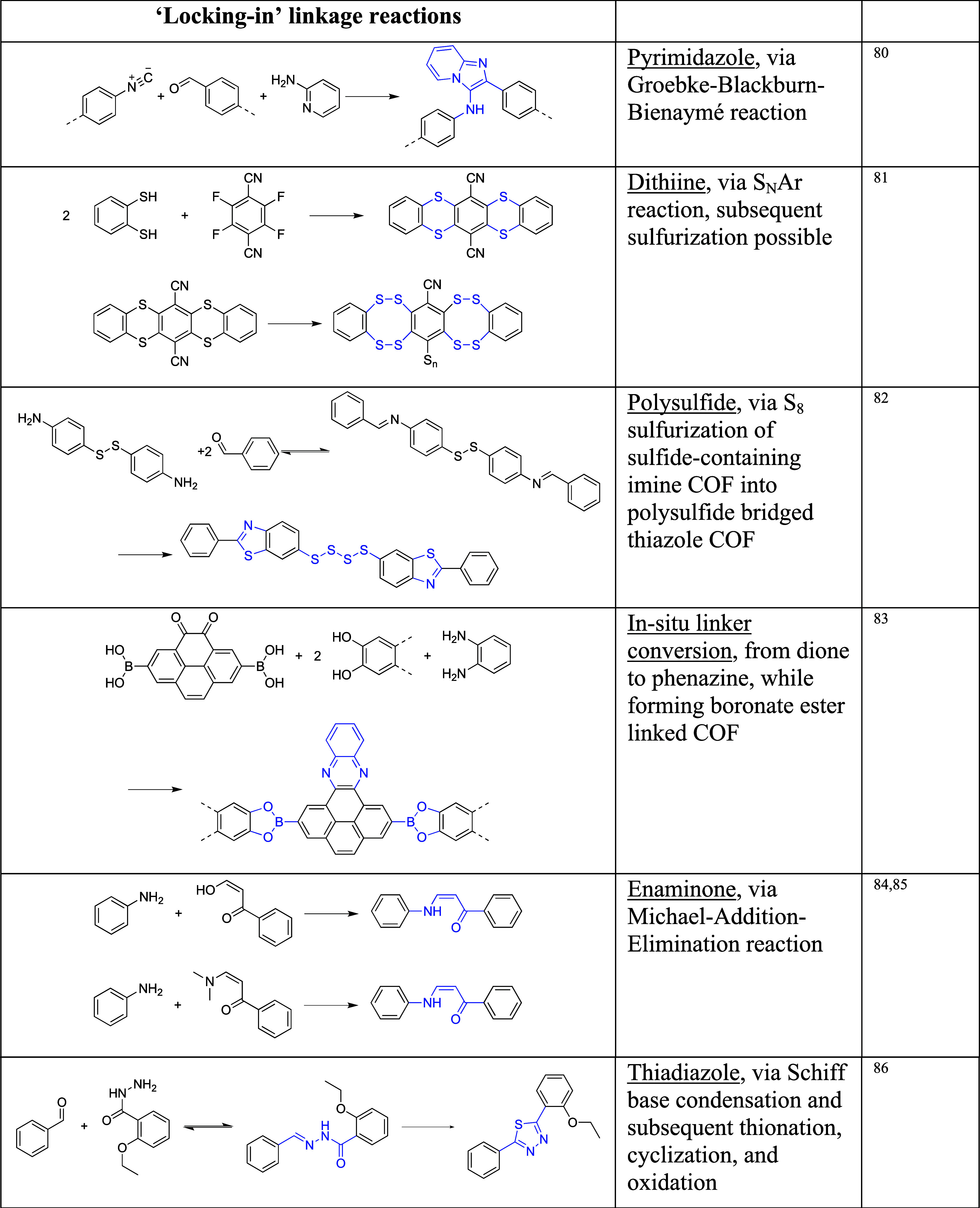
Commonly Used Linkage Formation Reactions and Locking-In Linkage Reactions of COFs, Illustrated with Model Reactions^a^,,,,,−

a Model reactions have been simplified for legibility. Curved arrows imply possible one-pot synthesis. Abbreviation: Ar = aromatic moiety.

In addition to linker chemistry, the type of energy input can have profound effects on the synthetic pathways,^55^ while multicomponent reactions can provide access to novel COFs in atom-efficient ways,^56^ and organic flux synthesis can be performed without additional solvents.^57^

### Stacking Patterns and Isomers

In addition to the linkage chemistry, the interactions of the 2D polymeric layers also have a profound impact on the electronic properties of the COFs. Different stacking patterns have been observed,^87^ the most dominant being the (nearly) eclipsed stacking of the aromatic polymer systems,^88^ while also serrated^89^ and staircase stacking^90^ have been discovered, for instance, when creating porphyrin-based COF layers,^91^ and in some cases, staggered stacking is also prevalent.^92^ It has been proposed that a certain degree of disorder in the stacking between the layers can be a dominant feature of many COFs.^93^ The layer stacking in COFs can also be directed by means of specifically selected nodes,^94,95^ via hydrogen-bonding^96^ or side-chain interactions,^97^ using ionic liquids,^98^ or by changing synthesis conditions,^99^ sometimes leading to well-defined interlayer interactions with unique minima in the energy hypersurface between the layers. This includes propeller-like node structures, such as tetraphenyl ethylene^100^ or dibenzochrysene^101^ building blocks. Moreover, isomeric COFs sometimes exhibit different structures,^102^ while the intermediate formation of macrocycles can lead to highly crystalline stacking.^103^ In this context, recently large language models (artificial intelligence, AI) were employed to optimize the crystallinity of framework materials through data science strategies and Bayesian optimization, where diverse AI collaborators served to assist in strategy planning, literature search, coding, robotic operation, labware design, safety inspection, and data analysis.^104^

**Figure 3:**
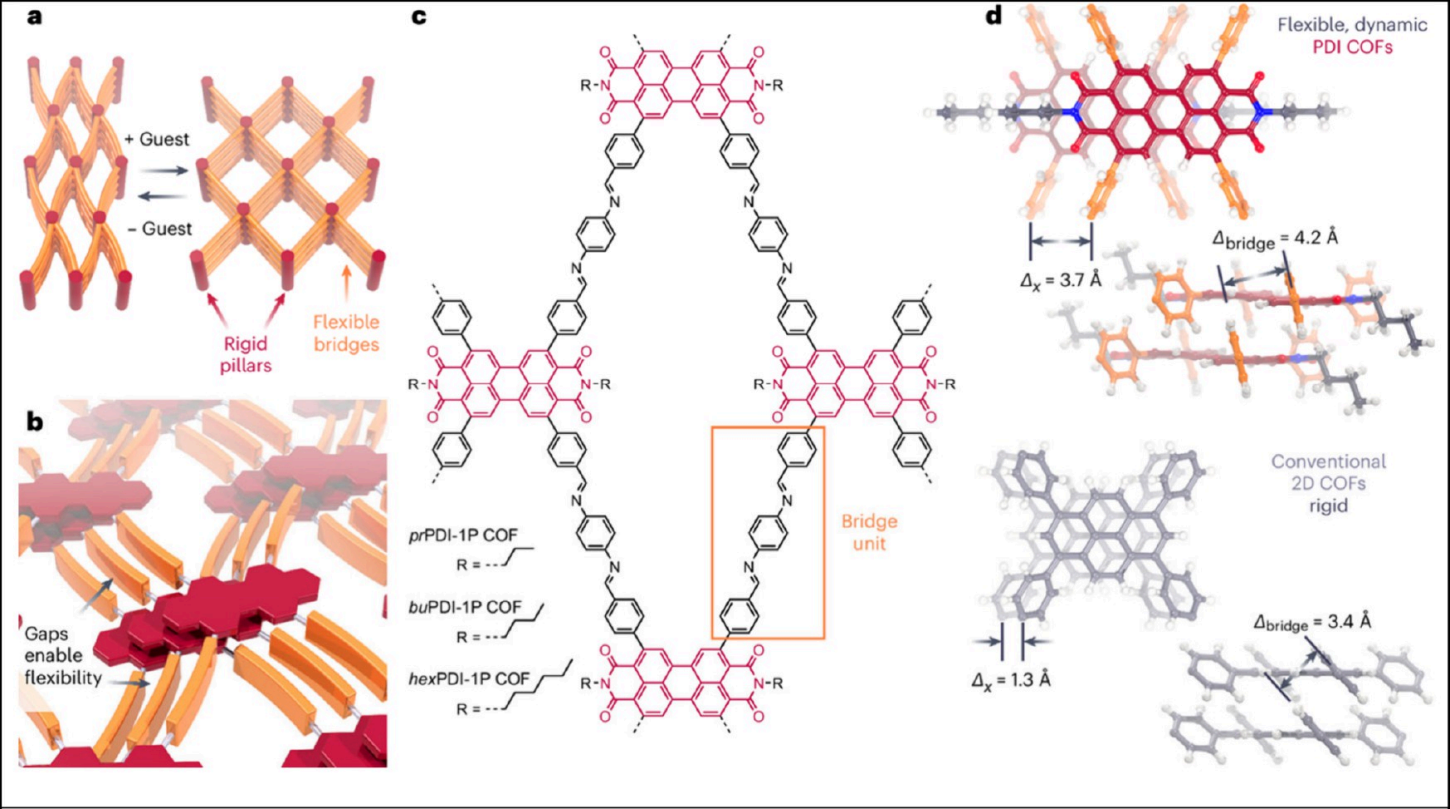
Construction of dynamic 2D COFs. (a) “Wine rack” like dynamic transformation, with (b) requiring a partially flexible framework of rigid pillars (red) interconnected via flexible bridges (orange) that are structurally prevented from forming close π–π contacts. (c) Chemical structure of the dynamic COFs, with (d) cut-outs of the new *bu*PDI-1P COF (top) and conventional pyrene COFs of similar topology (bottom), illustrating the different packing. In the PDI COF, the butyl substituents and ketones enforce a substantial lateral offset with large bridge-to-bridge distances ensuring flexibility. In contrast, the pyrenes stack with only small lateral offset, with rigid bridge units in close π–π contact. Reproduced with permission from ref (105). Copyright 2024 the author(s) of ref (105), under exclusive license to Springer Nature.

While a number of metalorganic frameworks (MOFs) show dynamic behavior upon guest adsorption, this phenomenon has been rarely reported for COFs. In recent work, 2D COFs were constructed that can dynamically switch between contracted and open-pore phases, based on a “wine-rack” motif with π-stacked perylene diimide (PDI) columns and flexible linkers (Figure 3).^105^ Intriguingly, these phase transformations have a strong impact on the optoelectronic properties of the COFs.

### Controlling Domain Sizes and Introducing/Understanding Defects.^106^

The crystalline domain sizes of COFs can be controlled by influencing nucleation rates, for instance, through slow monomer addition^107^ or the use of so-called modulators in the course of the crystallization.^108^ Modulators such as monovalent aldehydes or amines (in the case of imine condensation) or catechols (in the case of boronate ester linkages) compete with building blocks in the nucleation and crystal growth and have a profound influence on domain sizes.^109^ This approach was also demonstrated for the growth of large-domain 3D COFs.^110^ Modulators can also have a strong impact on the density of defects in the COF lattice,^111^ and passivation strategies for terminal reactive groups such as amines have been devised.^112^ On the other hand, defects can also be introduced intentionally,^113^ by changing stoichiometry,^114^ or by substituting a certain fraction of the building blocks with other building blocks (also called multivariate COFs,^115^ for instance, controlling porosity,^116^ tuning local charge distribution (and photocatalytic activity) by combining electron-donating and withdrawing groups in the pores,^117^ or implementing “truncated” nodes having fewer connection points for the generation of the lattice,^118^ resulting in nonconnected locations or additional functionality in the pores.^119^ Strain introduced by different building blocks can have a profound impact on COF formation.^120^

### Postsynthetic Modification

Another possibility to control the properties of COFs is by modifying the lattice after the initial synthesis.^121^ This vast field, also discussed in several earlier reviews,^4,5^ includes adding new functionalities via chemical grafting or changing the linkage by additional reactions, for instance, creating thiazole, oxazole, or thiazolothiazole linkages through postsynthetic reactions discussed above, through multicomponent Ugi reactions,^122^ or strongly modifying the electronic structure of the COF backbone.^123^ Moreover, even a complete exchange of (linear) linkers, for instance, by differentially functionalized linkers through linker exchange is a possibility to modify a COF after synthesizing the lattice. Examples include formation of amide COFs,^124^ transforming imine-linked COFs into their azo-linked analogs,^125^ asymmetric monomer exchange,^126^ and even dynamic transformations between COFs and discrete cages.^127^ An profound way of changing a COF’s crystal structure is by creating multivariate COFs where a broad selection of substitutable building blocks is mixed in the crystal lattice, resulting in the tunable density of functionalities such as ionic groups,^119b^ solid solutions,^128^ varying ratios of beta-ketoenamine to imine moieties in linkages,^129^ and possibly “alloy” formation of the optoelectronic properties. This general strategy opens up a vast range of tuning opportunities for these physical properties.

**Figure 4:**
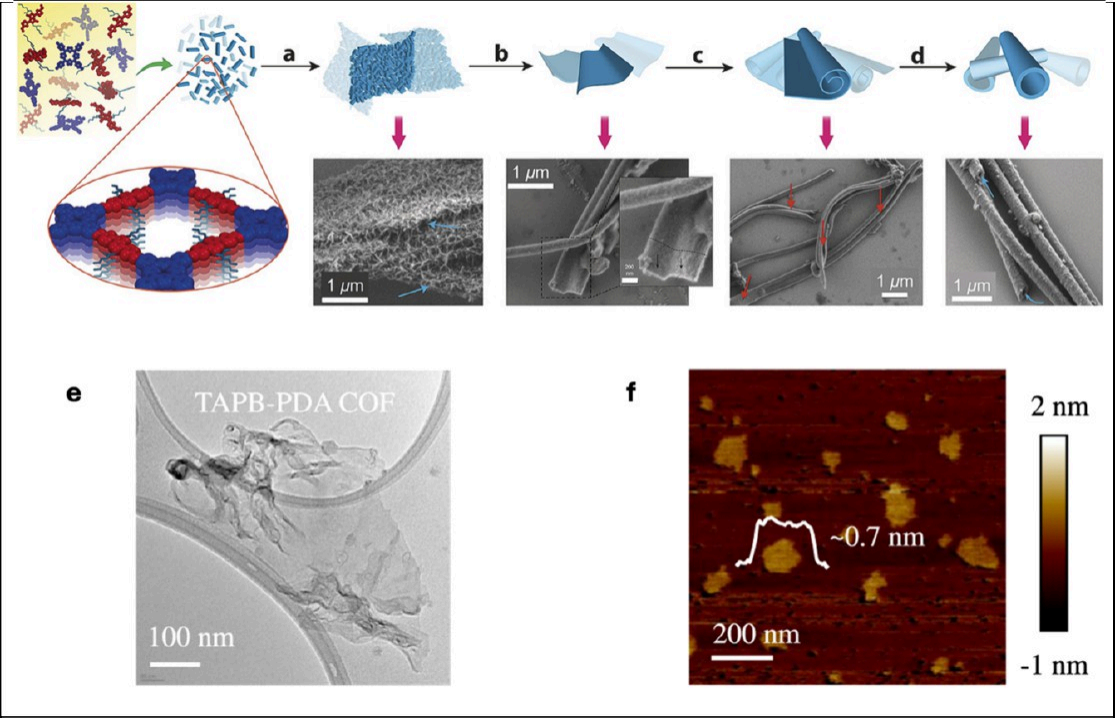
Nanoscale COF morphologies. Illustration of a proposed mechanism for microtube self-assembly of DPP-TAPP-COF with (a) agglomeration of small crystallites into sheet-like agglomerates, (b) smoothing and densification via continued reversable condensation reactions, (c) sheet rolling, and (d) tube formation and fusing via condensation reactions with corresponding SEM images. Reproduced from ref (10a). CC BY-NC-ND 4.0. (e, f) TEM and AFM images of exfoliated TAPB-PDA COF nanosheets, with inset showing height profile of a nanosheet. Adapted from ref (133a). CC BY 4.0.

**Figure 5:**
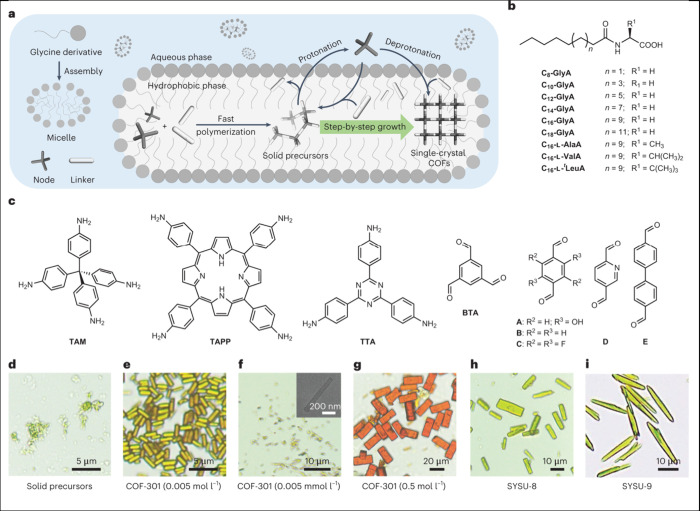
Growth of single-crystal imine-linked COFs in the reaction space of amphiphilic amino-acid derivatives in water. (a) Schematic view of methodology, using palmitoylglycine micellar compartments for the growth of the COFs. (b) Structures of the amphiphilic amino-acid derivatives. (c) Chemical structure of building blocks. (d–i) Optical microscopy and SEM images of (d) the solid precursors and (e–i) the synthesized products using C16-GlyA in water under ambient conditions: (e–g) COF-301, TAM, and linker A (shown in c); (h) SYSU-8, TAM, and linker C; (i) SYSU-9, TAM, and linker D. Reproduced with permission from ref (142). Copyright 2023 the author(s) of ref (142), under exclusive license to Springer Nature.

The ***morphology*** of COFs has a profound impact on many properties.^130^ While we will not discuss these features in detail, we note that COF nanoparticles (NPs)^131^ and their superstructures,^132^ nanosheets^133^ (see also Figure 4), fibers,^134^ COF-based tubes^135^ (also via COF-to-COF transformations),^136^ COF-related nanotubes,^137^ hollow structures,^138^ monoliths^139^ and thin films (see below) have been developed and can be adapted toward desired functionalities. Exciting progress has been made regarding the development of growth strategies for COF single crystals,^140^ and in controlling nucleation and growth through machine-learning.^141^ For example, a novel amino acid based micellar reaction space was devised to generate imine COF single crystals under mild conditions (Figure 5).^142^ Single crystals are not only of great value for structure determination and -evolution,^143^ but they also allow for reliable studies regarding optoelectronic and transport properties without being limited by the impact of grain boundaries.

### 3D COFs

**Figure 6:**
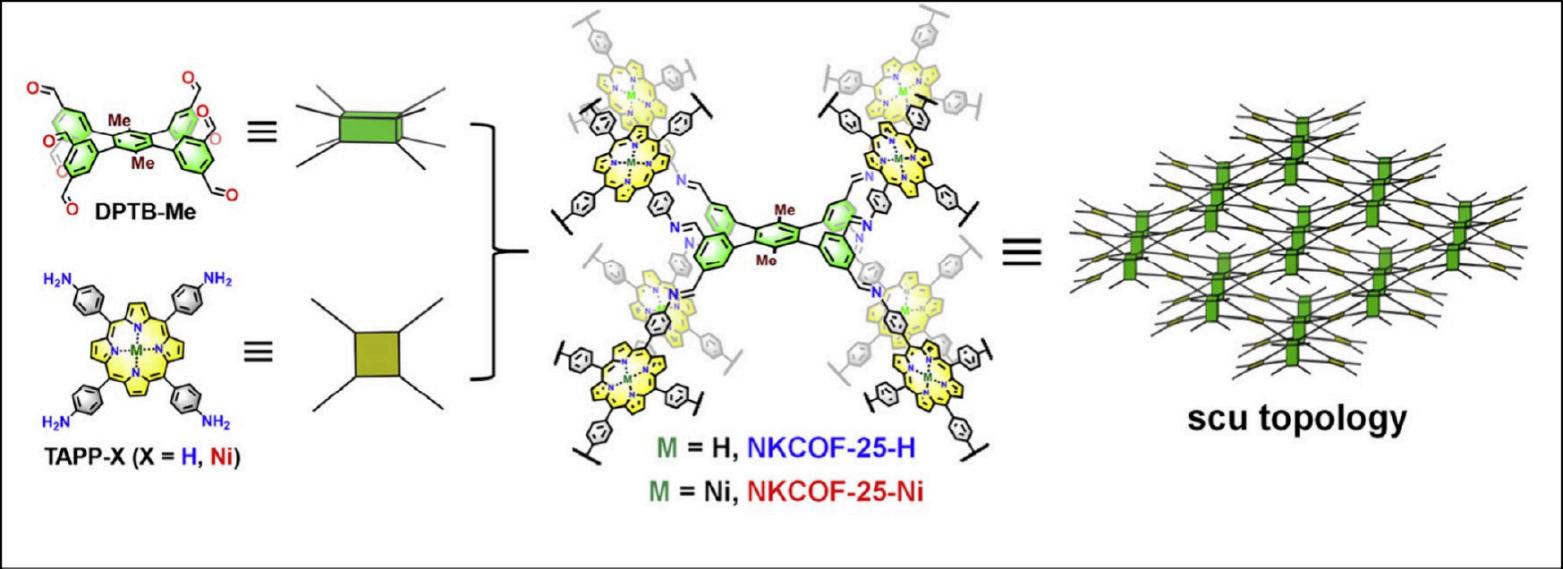
Construction of two 3D COFs featuring noninterpenetrated scu topology from an octatopic linker and porphyrin-based tetratopic linkers using an [8 + 4] approach. DPTB-Me is an 8-connected 3D-*D*_2*h*_-symmetric building block, and TAPP-X is a 4-connected 2D-*D*_2*h*_ monomer. Reproduced with permission from ref (153). Copyright 2022 Elsevier.

It is one of the major achievements of COF chemistry to be able to extend the structural design from two-dimensional to three-dimensional frameworks (some examples have been cited above), using appropriate molecular building blocks,^144^ such as those based on tetraphenyl methane (***dia*** topology).^27a,145^ The emerging panopticum of accessible topologies involves building blocks with tetrahedral, triangular, square, and even cubic-type shape or connectivity. Recent topology examples include ***ctn***,^146^***pcb***,^147^***crb***,^148^***scu***,^149^***shp***,^150^***qtz***,^151^***stp***,^152^ another, noninterpenetrated ***scu*** net from an octatopic node and a porphyrin-based tetratopic node through an [8 + 4] condensation (Figure 6),^153^***bcu***,^154^***pts***,^155^***nbo***,^156^***tbo***,^157^ homochiral porphyrin-frameworks with noninterpenetrated ***qzd*** topology,^158^ nets emerging from condensation of boronophenylphosphonic acid creating a phenyl-bridged network of cubes,^159^ and triptycene-based ***ceq*** or ***acs***(160) and ***lnj***(161) topologies, the latter being constructed from 12-connected triptycene nodes. Even 16-connected nodes have been integrated into 3D COFs.^162^ Control of interpenetration is of particular interest in this context.^163^ Given the demanding synthetic requirements for constructing three-dimensional crystalline frameworks through dynamic organic chemistry, specialized strategies have been developed, including the chemical reconstruction of a 2D COF to a 3D COF via base-triggered conversion of trigonal boronate to tetrahedral spiroborate linkages,^164^ processing in 3D reaction-diffusion zones,^165^ cross-linking diacetylene moieties,^166^ and postsynthetic annulation of hexaphenyl-triphenylene nodes to generate π-conjugated hexabenzo-trinaphthylene nodes in an ***she*** net with drastically changed optoelectronic properties.^167^

Recently the typical sp^3^ σ bond connections within 3D building blocks have been transcended, by employing the saddle-type structure of cyclooctatetrathiophene simulating a tetrahedral building block,^168^ and extending to sp^2^-carbon linkages in a 3D conjugated COF featuring a ***dia*** net and high charge-carrier mobility.^169^ Distorted polycyclic aromatic hydrocarbons can serve as triangular antiprismatic nodes in a 3D COF with a ***pcu*** net featuring favorable charge transport due to extended π-interpenetration,^170^ while metal complexes can also serve as COF nodes.^171−173^

### COF Thin Films and Membranes

**Figure 7:**
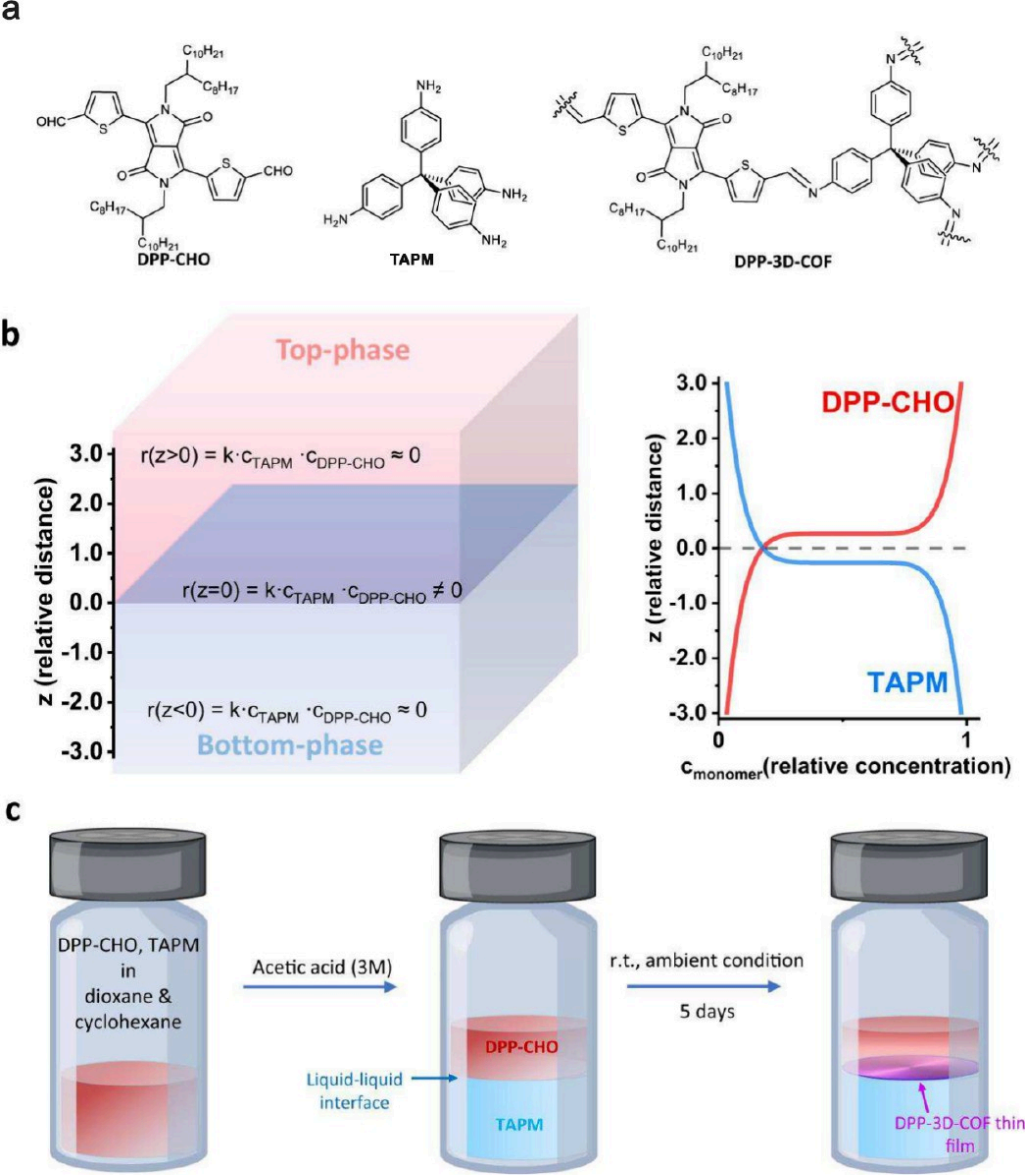
Generating a self-standing 3D COF film. (a) Chemical structures of the building blocks hydrophobic diketopyrrolopyrrole derivative (DPP-CHO), a hydrophilic tetrapod-amine (TAPM), and of DPP-3D-COF. (b) Reaction kinetics between DPP-CHO and TAPM in liquid–liquid solvent system (top: cyclohexane and dioxane, bottom: aqueous acetic acid and dioxane). At equilibrium, [TAPM] in the top phase is near zero and [DPP-CHO] in the bottom phase is near zero. (c) Liquid–liquid interfacial synthesis scheme for fabrication of DPP-3D-COF thin films (13 nm thickness). Adapted from ref (197). CC BY 4.0.

Thin films of COFs are of exceptional interest for numerous applications in fields such as photovoltaics, chemical sensors, lighting, or photo- and electrocatalysis, and have been grown on diverse types of substrates since many years. Overviews are given in the above-cited general reviews on COFs. It is possible to grow COF films in the same solvothermal synthesis environment as the bulk COFs,^21a,174,175^ provided an appropriate substrate is immersed in the synthesis solution (solid–liquid interface). Many examples of COF thin films, and even oriented COF thin films^176−178^ have been developed along this route. The control of crystal orientation can be improved by using highly ordered substrates such as graphene,^179^ or by solvent vapor annealing.^180^ Additional synthesis techniques include vapor-assisted conversion of precursor-layers on substrates,^181^ electrophoretic deposition of COF NPs,^182,183^ growth in a flow field,^184,185^ employing an electrogenerated acid^186^ or self-assembled monolayers,^187^ chemical vapor deposition (CVD) of small-molecule monomers,^188^ and spray coating.^189^ Layered 3D COF films could be grown through surface reactions of ionic B-based and neutral C-based COFs with tetrahedral nodes featuring similar unit cell parameters, thereby enabling (nearly) epitaxial stacking.^190^ Moreover, reactive suspensions such as COF nanoplatelets,^133b^ organic cages,^191^ COF seeds^192^ or amorphous COF gel spheres^193^ can serve as suitable precursors for COFs, be deposited on substrates and further converted into consistent COF films. Exciting recent studies have demonstrated the growth of free-standing thin films at liquid–air^194^ (e.g., surfactant-monolayer-assisted interfacial synthesis) or liquid–liquid interfaces (Figure 7),^195−197^ where the diffusion of reactive building blocks toward the immiscible liquid–liquid interface provides the synthetic environment for the growth of the COF thin films. Another approach toward forming extremely thin films is exfoliation of bulk 2D COFs and depositing the resulting COF nano sheets on substrates by solution processing techniques^198^ such as spin coating. Obviously, in the latter case a structural correlation of the individual COF layers in the final deposited thin film is not necessarily guaranteed, while repolymerization of the nanosheets can create consistent crystalline films. A key motivation for developing COF deposition methods beyond direct crystal growth is the expected acceleration and scalability of the deposition process.

## Fundamental Analysis of Electronic and Optoelectronic Properties

**Figure 8:**
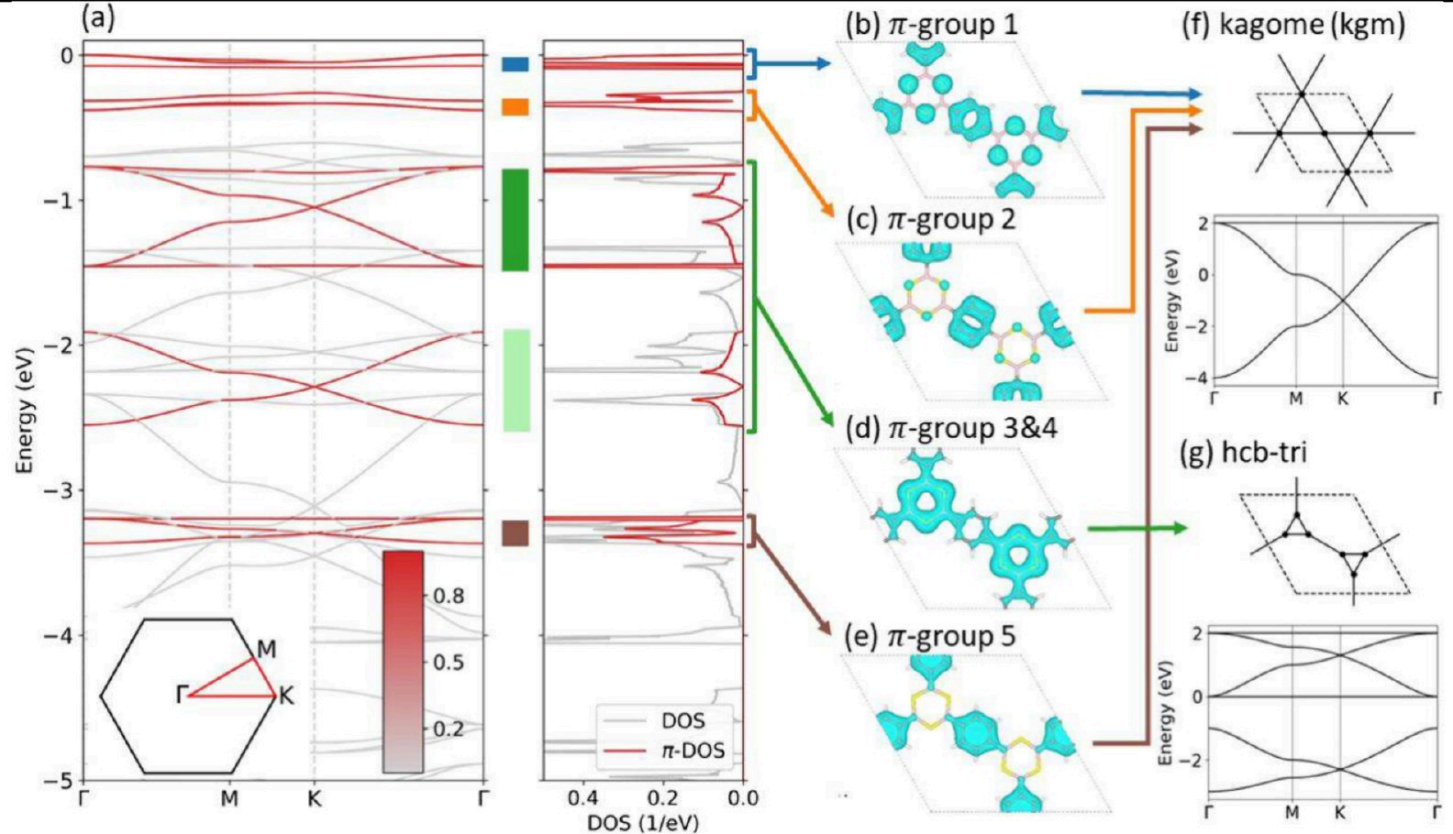
Modeling the electronic π-system of 2D COFs with DFT and Wannier functions, which enables the introduction of lattice models. (a) Band structure of COF-BS-1Ph in the valence band region and for monolayer geometry together with density of states (DOS) for all states and π-states (π-DOS). Projection on all π-orbitals is indicated as red shade. (b–e) Partial charge densities. (f, g) Comparison to the 2D effective lattice models. Reproduced from ref (199). CC BY 4.0.

The rich structural features of COFs discussed above can have a profound effect on their electronic band structure, their optical and charge-carrier transport properties and may enable functionalities in dedicated devices. The electronic properties of the building blocks, such as the size of a conjugated molecular moiety or donor–acceptor features within the building block on the one hand, and the nature of the linkage, i.e. the polarity of the linkage or the degree of electron delocalization through the linkage^199^ on the other hand, can have a strong effect on the COF’s electronic structure (Figure 8). This includes the value of the band gap, the occurrence of flat bands^200,201^ and the nature of the band gap, be it direct or indirect. Fundamental issues related to these features will be discussed in the following sections.

Besides their importance for many functionalities and applications, these electronic properties of 2D COFs have also attracted fundamental interest due to the interplay of different interactions, namely covalent interactions within the 2D layers and π–π interactions perpendicular to the layers, suggesting a strong electronic anisotropy. Knowing both the specific 2D electronic properties and the influence of interlayer interactions is therefore of great importance, but they are not yet well understood. Moreover, the electronic coupling through bonds in 2D COFs, within the planes (“in-plane coupling”), and the through-space electronic coupling, originating from π-stacking normal to the planes (“out-of plane coupling”), will influence not only the band structure but also the charge carrier transport pathways in the COF. It is of particular interest to learn about in-plane and out-of-plane charge carrier transport and enabling factors for high charge carrier mobilities.^202−206^ Moreover, the electrostatic potential within COF pores can also be tuned, for example by means of quadrupolar interactions.^207^

### Studies of Electronic Properties of 2D COF Systems

Cyclic voltammetry is a basic method that provides redox potentials for molecules and molecular thin films.^69,78,208−212^ Another technique to measure these potentials is photoemission. Here, ultraviolet photoelectron spectroscopy (UPS) provides information about the occupied density of states and, complementarily, inverse photoelectron spectroscopy (IPES) probes the unoccupied density of states. For films, however, photoemission studies or angle-resolved photoemission that could provide insights into electronic excitations, band structures and vibronic properties, such as for other 2D inorganic or organic systems^213−215^ are rare (here for both 2D polymer layers and COFs),^216−219^ thus calling for insights from theoretical and computational approaches.^220^

**Figure 9:**
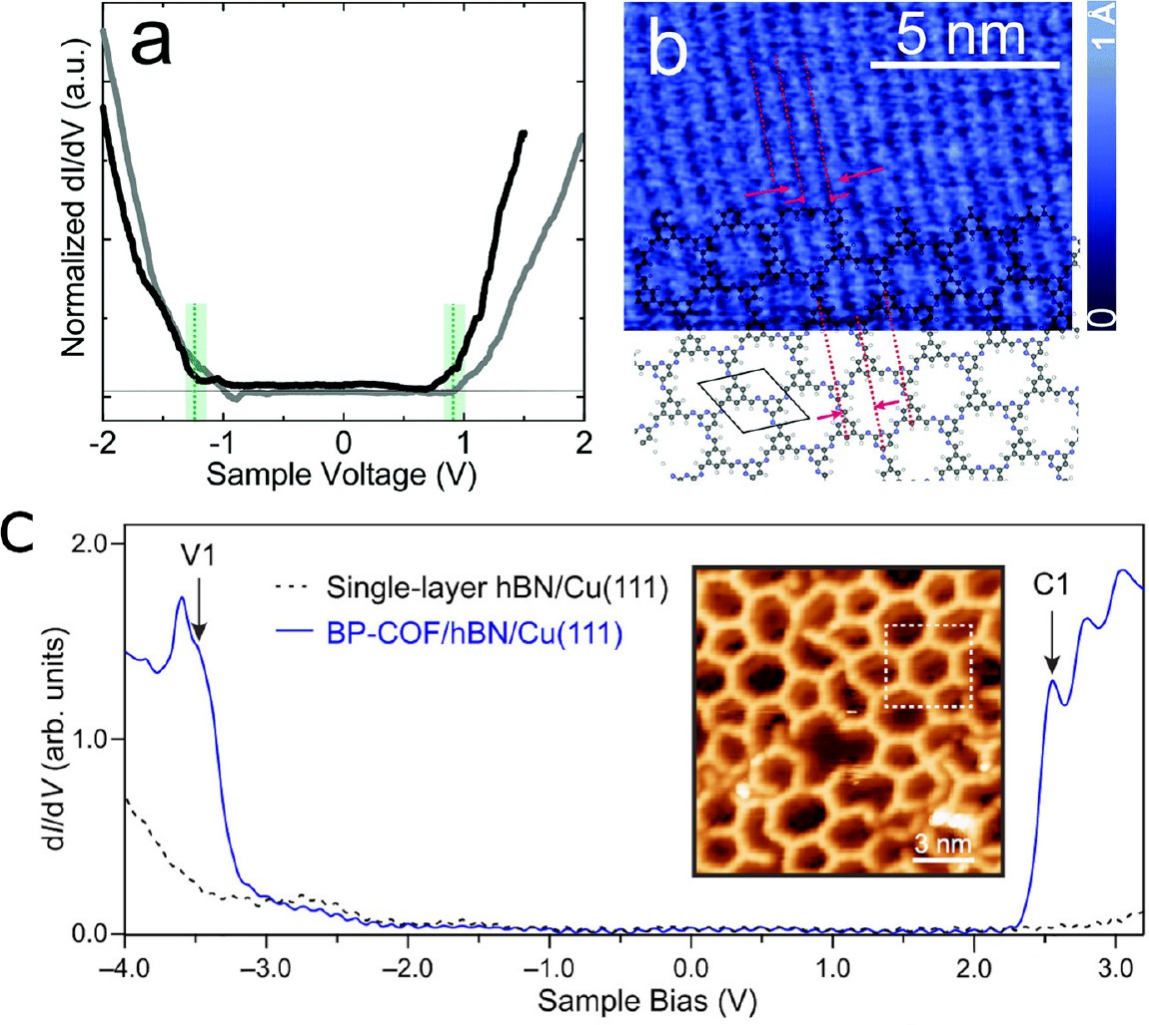
(a) Differential conductivity (d*I*/d*V*) obtained on 2D 2N-COF at the HOPG–air interface. Black and gray curves represent two independent sets of averaged measurements. (b) Constant current STM topograph of 2D 2N-COF on HOPG. Adjacent molecular rows in 2N-COF are marked by red dashed lines and compared to a DFT optimized geometry. (c) d*I*/d*V* point spectrum of BP-COF supported by hBN/Cu(111) showing both valence band edge (V1) and conduction band edge (C1) peaks. Inset: Topographic image of BP-COF/hBN/Cu(111). Reproduced with permission from (a, b) ref (223) and (c) ref (224). Copyright 2020 The PCCP Owner Societies and American Chemical Society, respectively.

Scanning tunnelling spectroscopy (STS) can provide complementary insights into the electronics of monolayer or few-layer assemblies with high spatial resolution. Besides several reports of successful microscopy images of COFs on (electronically) interacting metal substrates,^208,221,222^ a few studies achieved a certain degree of electronic decoupling of the COF from the metallic surface. For example, Yadav et al.^223^ used highly oriented pyrolytic graphite (HOPG) as an interlayer (Figure 9). To measure the density of states of the semiconducting 2D COF, the interlayer contributions to the dI/dV spectra of STS (the differential conductance) was subtracted in the gap region from the one part of the combined system (2D COF and HOPG). In another study, boroxine-based 2D COF structures were even more electronically decoupled from the metal support (in that case a Cu(111) surface) by employing a hexagonal BN (hBN) interlayer. This electronic 2D COF–surface decoupling allowed for investigating the monolayer properties of the COF, and its density of states was studied by STS.^224^ Of particular interest in STS studies is the electronic band gap. This energy gap between valence and conduction band states (also known as fundamental band gap or quasiparticle gap) is accessible through the frontier electronic states, which appear as resonances in the d*I*/d*V* spectra at high spatial resolution, which is inaccessible to photoemission. In this way, a quasiparticle band gap of 5.79 eV was determined for the COF/hBN/Cu(111) system^224^ in which the COF was a boroxine-linked biphenyl-based structure with a Kagome lattice. This large gap value reflects the small molecular building blocks (boroxine, biphenyl) and weak conjugation that makes this system an insulator. The specific samples studied there were not ideal in that they exhibited a large concentration of defects. The influence of defects such as grain boundaries and their characterization for COFs in general is an important task for future research.^225^ It has been proposed that systems different from boroxine-based materials can be semiconducting or even metallic, which triggered the quest for more strongly conjugated systems such as imine linked or C=C bonded COFs.

### Theoretical Analysis of Electronic Structure and Topology

Electronic properties such as redox potentials (charging energies) or photoemission spectra can be simulated using density functional theory (DFT).^226,227^ DFT approximates the electron–electron interaction at an ***effective-single-particle level*** and thus provides a very good compromise between accuracy and efficiency (see further discussion below) rendering it the theoretical workhorse for investigating COF materials, including the description of their electronic band structure and effective masses. It has been employed for 2D COFs with different lattices and is feasible for systems featuring small to very large unit cells^202,207,228−231^ and large building blocks such as phthalocyanines^232,233^ or hexabenzocoronene.^206,234^

The effective mass for electrons and holes can be understood as a result of the interplay of the electronic transfer integrals, i.e. the coupling between the molecular orbitals that are associated with the molecular fragments of the COF. For 2D COFs, this includes the coupling between orbitals in the 2D plane/layer that may lead to a Dirac cone, Kagome bands or other specific features (cf. Figure 8), and the coupling between the planes. The precise shape of the electronic band structure and the effective mass value result from a multitude of involved orbitals, their orbital energies and their transfer integrals between them. The number of possible combinations is too large to be discussed here in detail, but two main structural factors should be mentioned. A strong interlayer coupling (π-stacking) for instance leads to a strong band curvature in this direction and a reduced effective mass, whereas an increased effective mass is expected for increased layer distance. Independently, a lateral offset between the layers tends to reduce the electronic coupling between adjacent planes because of noncofacial stacking. Another effect relevant for COFs is the bond torsion that may disturb the in-plane conjugation and increases the effective mass. This can be avoided by ladder-type structures.

Intriguing features in the band structure such as ***Dirac cones*** (DCs) with a linear band dispersion and effectively massless electrons such as in graphene^235−238^ or at surfaces of topological insulators^239,240^ are being searched for in COFs as well. They have been discovered in theoretical studies on hexagonal COFs made from small oligomers (e.g., polythiophene, polyacetylene, polypyrene, polypyrrole, poly(p-phenylene)) connected in a hexagonal network via trivalent carbon atoms serving as nodes,^241−243^ reminiscent of the triphenylmethyl radical,^244^ as well as benzene- and benzotrithiophene-derived nodes. For these nodes in hexagonal COFs, DCs can occur at the Fermi level. Moreover, DCs can frequently occur in different energy regions of the bands.

The electronic bands can be further classified in the context of band topology,^245,246^ which is closely related to the Berry phase physics of these structures. Both are also directly connected to measurable quantities (observables) such as charge conductivity and may result in specific features. First steps in the prediction of such structures include triangulenes, which can form Dirac semimetals.^217,247^ ARPES data on the electronic properties of such structures, in which the vertex is made, for example, from tribromotrioxaazatriangulene, show a pair of Dirac bands for a honeycomb lattice.^217^ This observation was enabled by the mesoscale size of the domains on the Au surface on which they had been polymerized. In addition, a two-dimensional topological Dirac semimetal phase was predicted in a strained tetragonal phthalocyanine-based COF.^248^ Other types of topological classifications have also been transferred to COFs.^249^

The anisotropic structure of 2D COFs has also motivated the investigation of unusual topological states. For example, Lieb lattices, based on triangular building blocks featuring suitable electronic coupling, possibly lead to interesting electronic behavior such as a Dirac-flat band structure.^250^ The authors predict that two configurational COF isomers based on reported sp^2^-carbon linked tetraphenylpyrene are distorted Lieb lattices, opening the possibility to design organic spintronic devices.

A band feature that has lately gained considerable interest, in analogy to few-layer graphene, is the presence of flat bands. Flat bands are, for instance, induced in twisted bilayer graphene.^251−255^ Intriguingly, flat bands imply an infinite effective mass *m* according to a vanishing band curvature , which is opposite to the case of DCs where the band curvature vanishes as well, but the energy dispersion *E*(*k*) is linear as for massless Dirac Fermions.^235^ This raises the question of why flat band cases would be of interest. One reason is that electron–electron interaction is a long-standing and very challenging research target. This interaction induces quantum correlations that are interesting to study in general—not only but specifically in periodic systems. The presence of additional energy terms that compete with the electron–electron interaction, such as the kinetic energy, which often entails a large bandwidth, gives rise to new phenomena and new phases but makes theoretical studies of correlation phenomena a challenging problem. At vanishing bandwidth, the kinetic energy drops effectively to zero, opening a complementary regime for investigations of electron correlations.

### Comparison of Computational Approaches to Electronic Structure

DFT is, besides its successful predictions of many properties, rather inaccurate in predicting the absolute (energetic) positions of the electronic bands of semiconductors if, as is often the case, local or semilocal exchange-correlation (XC) functionals (e.g., LDA, PBE, PW91, BLYP etc.)^256−259^ are used and the Kohn–Sham energies are interpreted as single-particle excitation energies (redox potentials) of the system. This general “failure” is known from inorganic semiconductors^260,261^ and persists in COFs as well as in gas-phase molecules. As a result, the band gap energies are severely underestimated by such local or semilocal XC functionals^262^ and might even suggest a metal for what is actually a semiconductor.^263^

A way to circumvent this issue is the use of hybrid XC functionals (such as HSE^264,265^ B3LYP^266^ or PBE0^267^ that can improve this situation. In semiconducting 2D COFs, the band gap opening obtained with hybrid functionals^228^ was almost a factor of 2 compared to the semilocal PBE functional.^223,268^ Another systematic way to improve the calculated energetic position of the electronic bands is to employ many-body perturbation theory,^269,270^ in which the electron–electron interaction is described more accurately. The most common approach is the so-called GW-approximation,^271^ which has been applied to obtain more accurate quasiparticle energy gaps in COFs.^201,272,273^ While this yields an improved description of electronic interactions at the single (quasi-)particle level, similar to molecules^274^ and two-dimensional systems,^275^ simulating optical properties or neutral (excitonic) excitations requires a genuine two-particle picture.^276^ In general, these excitations can be of interband type or bound electron–hole pairs, so-called excitons, which are manifested in peak-like subgap absorption features in semiconductors.^276^ The Coulombic attraction between electron and hole is responsible for the lower optical gap as compared to the above-mentioned fundamental band gap. Indeed, such excitonic effects in optical spectra are expected to be strong because of the porosity of 2D COF materials that suggests low dielectric constants. An ultralow relative permittivity of κ = 1.6 has been measured for the insulating COF-5 by impedance spectroscopy.^277^ Low dielectric constants imply that the Coulomb interaction between electrons and holes is enhanced as compared to other layered materials.^278^ Moreover, this enhanced interaction (weak screening) is not restricted to excitons, potentially allows for an unprecedented tunability of electronic properties in analogy to other organic materials,^279−282^ and remains to be experimentally explored.

### π Conjugation and Modification of the Electronic Structure

The electronic structure of COFs is influenced by the electronic properties of the building blocks, e.g.,^12^ and theoretical work shows that the former can be tuned by chemical functionalization/modification of the latter.^283^ In addition, it is of interest to identify the impact of joining COF building blocks in the lattice on their electronic features. For example, the degree of π-conjugation and aromaticity of linkers within similar square-lattice topologies can have a profound effect on the band structure.^284^ Merkel et al. have studied a family of hexagonal COFs and found delocalized states that are spread across tens or hundreds of pores of the 2D COFs, depending on the linker moiety and disorder.^199^ In addition, in several sp^2^-carbon linked D-A COFs it was established that a noncoplanar framework can become more conjugated due to an increasing degree of charge transfer between core (node) and linker units during the construction of 2D frameworks.^285^ This can increase the charge carrier mobility within the 2D COFs.

The electronic structure of (imine-linked) COFs can also be indirectly modified by different relative orientations of the imine bonds in the lattice leading to geometric isomers, impacting the stacking of the 2D layers, and even influencing the crystallinity of the structures.^286^ The molecular understanding of interlayer stacking and its impact on electronic properties is an active domain of COF research.^287,288^

### AC Conductivities

Early flash-photolysis time-resolved microwave conductivity (TRMC) experiments with COFs^289−291^ have shown decent carrier mobilities of a few cm^2^ V^–1^ s^–1^.^292^ With this technique, electrons and holes are generated optically by a laser flash. Both contribute jointly to the ac conductivity signal and cannot be separately addressed, while in the case of different mobilities the more mobile charge carriers will dominate the response. Here, the mobility is a transient quantity describing the carrier’s response to rapidly fluctuating fields, where they oscillate forth and back and hardly cover any distance. The influence of conventional carrier trapping is reduced as compared to a macroscopic transport experiment possibly probing the material across different grains. Additionally, metallic contacts are absent in TRMC. These contact-less measurements yield an effective direction-averaged conductivity or mobility value at ultrahigh frequencies in samples that are (usually) randomly orientated and hence the results can be regarded as an intrinsic material property. Similarly, terahertz (THz) mobilities are usually measured for optically generated charges.^18^ These transient mobilities indicate a potential for good carrier transport. Recently, THz conductivity studies of 3D COF membranes have been reported, which however are based on surprisingly low calculated effective masses.^293^

### Charge Carrier Transport

In transport experiments, on the other hand, device-based carrier mobilities are obtained by involving electrical contacts. These devices and their carrier mobilities are closer to possible applications than microwave-derived mobility values of the materials. They can be measured in two- or four-probe setups or in field effect transistors (FETs). In such FET devices the current–voltage characteristics are directly accessed from linear or saturation regimes. Early work with COFs showed FET mobilities that were often much below the above-mentioned TRMC values. However, in recent years more and more examples have been demonstrated with device mobility values above 1 cm^2^ V^–1^ s^–1^, which is close to the mobility of amorphous silicon that is often cited as a reference value. The mobility values can also surpass the mobilities of small molecule organic crystals.^294,295^ A compilation of recently reported measurements is found in Table 2 below. Interesting observations have been made, such as an unexpected Hall effect in the presence of 1D electronic band dispersion in the stacking direction of the COF layers and flat bands in in-plane directions.^313^ They indicate that the charge transport through the π-conjugated system (and the transport anisotropy in general) remain to be fully understood. Observations of magnetotransport^296^ or direction-dependent charge transport also exist^210,297,298^ and need to be further explored to develop a comprehensive picture.

**Table 2:** Transport Properties of Different COFs^a^

chemical building blocks	mobility (cm^2^ V^–1^ s^–1^)	conductivity (S/cm)	*E*_A_ for conductivity (meV)	technique	dopant	comments	linkage motif	ref	year
dioctyloxybenzodithiophene, triphenylamine	3 × 10^–6^			FET	–		imine	(310)	2015
hexaaminobenzene, hexaketocyclohexane	6.5 × 10^–3^			FET (ave.)	–	annealed	pyrazine	(208)	2015
	1.3 × 10^–2^			FET (max.)	–	annealed	pyrazine	%	
	13.5			FET electron	unintentional	unannealed, (estimated)	pyrazine	%	
	20.6			FET hole	unintentional	unannealed, (estimated)	pyrazine	%	
tetraphenylporphyrin, dihydroxybenzene	1.3 × 10^–6^			FET	–		imine	(311)	2016
	1.6 × 10^–4^			FET	I_2_		imine	%	
triazine, benzene	1.5 × 10^–1^			FET	–	Single layer/multilayer	triazine	(312)	2017
TPPy, benzene				vertical FET	–	high current densities, ambipolar	imine	(305)	2017
ZnPc, bis(*tert*-butyl)pyrene	4.8		340 (vdP)	Hall mobility	–	p-type	pyrazine	(313)	2019
CuPc, bis(*tert*-butyl)pyrene	0.9		340 (vdP)	Hall-mobility	–	p-type	pyrazine	%	
ZnPc, bis(*tert*-butyl)pyrene	2			THz	–		pyrazine	%	
CuPc, bis(*tert*-butyl)pyrene	0.7			THz	–		pyrazine	%	
NiPc, pyrene		2.5 × 10^–5^	324	4-point	–		pyrazine	(314)	2019
				4-point	I_2_		pyrazine	%	
triphenylene	1.96			FET (ave.)	–	PXRD: broad in-plane diffractions	pyrazine	(78)	2020
	4			FET (max.)	–		pyrazine	%	
ZnPc, *tert*-butylpyrene	22		210 (Hall)	Hall-mobility	I_2_	p-type	pyrazine	(299)	2020
CuPc, *tert*-butylpyrene	7			Hall-mobility	I_2_	p-type	pyrazine	%	
ZnPc, *tert*-butylpyrene	6.3			THz	I_2_		pyrazine	%	
W, benzene		4.91 × 10^–8^		4-point (vdP)	–	pressed pellet	imine	(210)	2020
W, benzene		3.78 × 10^–8^			–	oriented film	imine	%	
W, benzodithiophene		2.70 × 10^–6^		4-point (vdP)	–	pressed pellet	imine	%	
W, benzodithiophene		1.64 × 10^–5^			–	oriented film	imine	%	
W, benzodithiophene		3.67 × 10^–2^			F_4_TCNQ	pressed pellet	imine	%	
W, benzodithiophene		3.09 × 10^–4^			SbCl_5_	pressed pellet	imine	%	
W, benzodithiophene		4.72 × 10^–4^			I_2_	pressed pellet	imine	%	
W, benzodithiophene		2.18 × 10^–2^			F_4_TCNQ	thin film	imine	%	
W, benzodithiophene		6.86 × 10^–4^			SbCl_5_	thin film	imine	%	
W, benzodithiophene		1.33 × 10^–4^			I_2_	thin film	imine	%	
W, benzene		1.51 × 10^–7^			F_4_TCNQ	pressed pellet	imine	%	
W, benzene		7.35 × 10^–7^			F_4_TCNQ	oriented film	imine	%	
hexahydroxytriphenylene, MIDA		6 × 10^–6^	300	vdP	–	electron transport in the nonconjugated COF	boronate ester	(302)	2021
CuPc, MIDA		3 × 10^–6^	340	vdP	–		boronate ester	%	
hexahydroxytriphenylene, MIDA				Hall-mobility	–	n-type	boronate ester	%	
CuPc, MIDA	8.3			Hall-mobility	–	n-type	boronate ester	%	
hexahydroxytriphenylene, MIDA	3.4			THz	–		boronate ester	%	
CuPc, MIDA	13.3			THz	–		boronate ester	%	
NiPc	0.15			Hall-mobility	–	p-type	Co-tetra-aza[14]-annulene	(315)	2021
NiPc		8.16 × 10^–5^	330	4-point	–	pressed pellets	CoTAA	%	
NiPc		6.67 × 10^–5^		current–voltage	–	films	CoTAA	%	
NiPc		5.2 × 10^–3^		4-point	I_2_	pressed pellets	CoTAA	%	
tetrafluoroterephthalonitrile, PcCo	19.4	1.9 × 10^–6^		Hall-mobility	–	p-type	dioxine	(211)	2021
tetrafluoroterephthalonitrile, PcCu	0.3	2.6 × 10^–7^		Hall-mobility	–	p-type	dioxine	%	
tetrafluoroterephthalonitrile, PcNi	3.2	9.3 × 10^–7^		Hall-mobility	–	p-type	dioxine	%	
tetrafluoroterephthalonitrile, PcCu		3.8 × 10^–7^		2-point	–		dioxine	%	
tetrafluoroterephthalonitrile, PcCu		2.0 × 10^–3^		2-point	I_2_		dioxine	%	
pyrene–hexaaminobenzene	996			FET	–	n-type	pyrazine	(303)	2021
pyrene–hexaaminobenzene	501			FET	–	p-type	pyrazine	%	
pyrene–hexaaminobenzene		7.2			–		pyrazine	%	
pyrene–hexaaminobenzene		1038			HCl	at high temperature	pyrazine	%	
phenylethynylanthracene, triformylphloroglucinol (AntTTH)			14.3 (FP-TRMC)	FP-TRMC, photoconductivity	–		enamine	(297)	2022
phenylethynylanthracene, triformylbenzene				FP-TRMC, photoconductivity	–		imine	%	
phenylethynylanthracene, AntTTH		3.0 × 10^–8^	229	2-point	–	dark	enamine	%	
phenylethynylanthracene, AntTTH		1.2 × 10^–7^	222	2-point	–	light	enamine	%	
benzotrithiophene-T3, triphenyltriazine		8.7 × 10^–6^	123.3	2-point, in plane	–		imine	(298)	2022
benzotrithiophene-T3, tris(biphenyl)triazine		1.1 × 10^–6^	137.5	2-point, in plane	–		imine	%	
benzotrithiophene-T3, tris(biphenyl)triazine		9.5 × 10^–13^		2-point, out-of-plane	–		imine	%	
diphenylanthracene, benzene		8.5 × 10^–4^	130	4-point	I_2_	pressed pellets	imine	(205)	2022
benzene, biphenyl		3.6 × 10^–5^		4-point	I_2_	pressed pellets	imine	%	
diphenylanthracene, benzene	0.7			Hall-mob	–	p-type	imine	%	
diphenylanthracene, benzene	10.5			Hall-mob	I_2_	p-type	imine	%	
benzene, biphenyl	4.3			Hall-mob	I_2_	p-type	imine	%	
TPB, benzene	165			THz	–	films, Drude-type band transport	imine	(304)	2022
TPB, diphenylnaphthalene diimide		<1 × 10^–9^		2-point	–	pressed pellets	imine	(219)	2022
TPPy, diphenylnaphthalene diimide		<1 × 10^–10^		2-point	–	pressed pellets	imine	%	
TPB, diphenylnaphthalene diimide		7 × 10^–5^		2-point	cobaltocene	pressed pellets	imine	%	
TPPy, diphenylnaphthalene diimide		6 × 10^–4^		2-point	cobaltocene	pressed pellets	imine	%	
TPPy, PDAN	22.1			THz	–		C=C	(316)	2022
(pyrene-2,7-diyl)bis(terphenyl), PDAN	2.3			THz	–		C=C	%	
(triphenylene-2,7-diyl)bis(terphenyl), PDAN	<0.1			THz	–		C=C	%	
(benzodithiophene-2,6-diyl)bis(terphenyl), PDAN	5.8			THz	–		C=C	%	
TPPy, PDAN	51.1			THz	I_2_		C=C	%	
(pyrene-2,7-diyl)bis(terphenyl), PDAN	5.7			THz	I_2_		C=C	%	
INDO4, TPB		8.27 × 10^–5^		2-point	–	pressed pellets	C=C	(317)	2022
INDO4, TPB		7.44 × 10^–3^		2-point	N-DMBI	pressed pellets	C=C	%	
NiPc,CoPcF8	23.7	2.72 × 10^–2^		Hall; 4-point	–	pressed pellets, p-type	piperazine	(204)	2022
NiPc,CuPcF8	20.2	2.41 × 10^–2^		Hall; 4-point	–	pressed pellets, p-type	piperazine	%	
NiPc,CoPcF8	35.4	1.27 × 10^–1^		Hall; 4-point	–	film, p-type	piperazine	%	
NiPc,CuPcF8	35.1	1.15 × 10^–1^		Hall; 4-point	–	film, p-type	piperazine	%	
NiPc,CoPcF8	0.23			FET	–	film, p-type	piperazine	%	
INDO4, TPPy		1 × 10^–3^		4-point	–		C=C	(11)	2023
INDO4, tetraphenylperylene		1 × 10^–3^		4-point	–		C=C	%	
INDO4, TPPy	7.8			Hall-mobility	–	compressed pellets, p-type	C=C	%	
INDO4, tetraphenylperylene	6.4			Hall-mobility	–		C=C	%	
carbazolylene-ethynylene, benzene		7.8 × 10^–4^	90	4-point	I_2_	pressed pellets	azine	(319)	2023
carbazolylene-ethynylene, benzene, biphenyl		3.4 × 10^–5^	460	4-point	I_2_	pressed pellets	imine	%	
BBL, NiPc	971			THz	–	films, Drude-type band transport	BBL	(18)	2023
BBL, CuPc	460			THz	–	films, Drude-type band transport	BBL	%	
BBL, NiPc	219			THz	–	powder	BBL	%	
BBL, CuPc	183			THz	–	powder	BBL	%	
TPPy, fluorenone	63.6			Hall-mobility	Ni	n-type	imine	(320)	2023
thienyl-benzodithiophene, bithiophene	65			THz	–	band-like	C=C	(16)	2023
tetraphenylethene, diphenylanthracene		1.4 × 10^–7^	125	2-point	–	p-type, vertical devices	imine	(321)	2023
tetraphenylethene, 2,5-dihydroxybenzene		1.3 × 10^–4^	63	2-point	–	p-type, vertical devices	imine	%	
pyrene-isoindigo	6.6	5.3 × 10^–3^		Hall, vdP geometry		n-type, amorphous PXRD	acetylene	(322)	2023
benzene-isoindigo	3.5	1.7 × 10^–4^		Hall, vdP geometry		n-type, amorphous PXRD	acetylene	%	
hexaazatriphenylene core, hexahydroxytriphenylene		1.46 × 10^–3^		2-point	–	film	dioxin	(323)	2023
hexaazatriphenylene core, hexahydroxytriphenylene		1.25	42	2-point	tris(4-bromophenyl)ammoniumyl hexachloroantimonate (p-type)	film	dioxin	%	
CuPc, anthraquinone	601.5	1.53 × 10^1^		Hall, vdP geometry	–	film	dioxin	(324)	2024
CuPc, anthraquinone	37.2	4.14 × 10^–1^		Hall, vdP geometry	–	pressed pellets	dioxin	%	
CuPc, 9,10-dimethyl-anthracene	367.3	1.07 × 10^1^		Hall, vdP geometry	–	film	dioxin	%	
CuPc, 9,10-dimethyl-anthracene	21.4	2.51 × 10^–1^		Hall, vdP geometry	–	pressed pellets	dioxin	%	
NiPc, anthraquinone	563.8	1.43 × 10^1^		Hall, vdP geometry	–	film	dioxin	%	
NiPc, anthraquinone	36.2	3.79 × 10^–1^		Hall, vdP geometry	–	pressed pellets	dioxin	%	
CoPc, anthraquinone	547.2	1.37 × 10^1^		Hall, vdP geometry	–	film	dioxin	%	
CoPc, anthraquinone	32.7	3.53 × 10^–1^		Hall, vdP geometry	–	pressed pellets	dioxin	%	
tetraphenylpyrene, diethyl oxybenzene-1,4-di(formylhydrazine)	0.12	3.3 × 10^–5^	220	Hall	I_2_	p-type	hydrazone	(325)	2024
tetraphenylpyrene, benzene	0.36	6.8 × 10^–5^	220	Hall	I_2_	p-type	imine	%	
tetraphenylpyrene	0.47	1.13 × 10^–4^	200	Hall	I_2_	p-type	azine	%	
tetraphenylpyrene, benzene	0.68	1.69 × 10^–4^	240	Hall	I_2_	p-type	C=C	%	
benzimidazole-CuPc, benzene		2.18 × 10^–3^		2-point	–	film	imidazole	(326)	2024
benzimidazole-CuPc, dimethoxy-benzene		2.2 × 10^–4^		2-point	–	film	imidazole	%	
benzimidazole-CuPc, bipyridyl		3.4 × 10^–4^		2-point	–	film	imidazole	%	
hexaone-trimesityl-triazacoronene, phenazine		1.1 × 10^–9^		vdP geometry	–	Broad reflections in XRD, pressed pellets	phenazine	(327)	2024
hexaone-trimesityl-triazacoronene, phenazine		2.5 × 10^–1^		vdP geometry	I_2_	Broad reflections in XRD, pressed pellets	phenazine	%	
hexaone-trimesityl-triazacoronene, phenazine	35.8			THz	–	Broad reflections in XRD, pressed pellets	phenazine	%	

a Chemical building blocks are presented in abbreviated form. Abbreviations: W = tetraphenyl-1,4-phenylenediamine, Pc = phthalocyanine, MIDA = methyl-isoindigo diboronic acid, TPPy = tetraphenylpyrene, TPB = triphenylbenzene, CoTAA = cobalt(II)tetraaza[14]annulene, BBL = benzimidazobenzophenanthroline, PDAN = 2,2′-(1,4-phenylene)diacetonitrile, INDO4 = s-indacene-1,3,5,7(2*H*,6*H*)-tetraone. vdP = van der Pauw. The symbol % refers to the same reference as above.

Higher mobilities and conductivities are typically found for doped systems, indicating that carrier transport is still trap-limited. This is consistent with the thermal activation behavior, leading to higher conductivities at higher temperature. The activation behavior in the doped systems is usually weaker with a lower activation energy of typically *E*_A_ = 100–300 meV.^299^ This range is still an order of magnitude larger than in doped molecular blends^300^ and it can be expected that even lower *E*_A_ values should further improve charge transport.^300^ An example of very low *E*_A_ and still moderately high conductivities of 10^–2^ S/cm was reported in an iodine-doped (aza)triangulene-based system suggesting a “bad metal” transport regime.^301^ Whether even higher conductivities with such systems could be observed with different doping methods is an open question. In the absence of doping, Jin et al.^302^ demonstrated that formally nonconjugated boronate linkages in hexagonal and tetragonal COFs can also allow for high electron mobilities.

**Figure 10:**
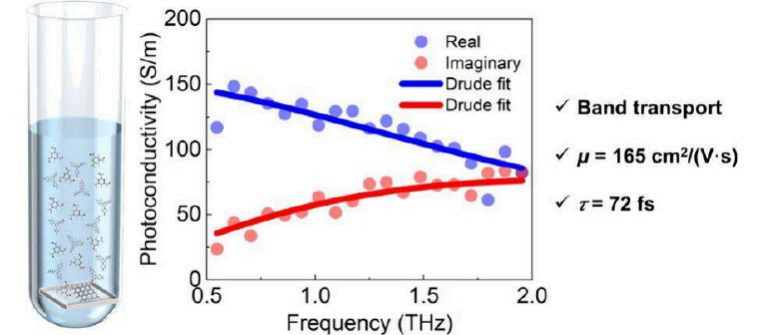
Frequency-resolved complex THz photoconductivity of a TPB–TFB COF thin film at 1 ps pump–probe delays following optical excitation. The blue and red dots represent the real and imaginary parts, respectively. Solid lines correspond to the Drude model. Reproduced from ref (304). CC BY 4.0.

Mahmood et al. fabricated field effect transistors (FETs) employing pyrene–pyrazine based^303^ frameworks featuring high mobilities of about 501 and 996 cm^2^ V^–1^ s^–1^ for holes and electrons, respectively. For the same material, doping-induced conductivities of up to 1038 S cm^–1^ were reported. For semiconducting 2D COF thin films obtained by polymerizing 1,3,5-tris(4-aminophenyl)benzene (TPB) and 1,3,5-triformylbenzene (TFB),^304^ (Figure 10) intrinsically Drude-type band-like transport (negative temperature coefficient ) was demonstrated for optically generated carriers probed with THz pulses, with very high charge carrier mobility (165 cm^2^ V^–1^ s^–1^),^304^ and THz mobilities of up to about 970 cm^2^ V^–1^ s^–1^ were found in benzimidazobenzophenanthroline ladder-type 2D COF films, which were further increased at lower temperature.^18^

**Figure 11:**
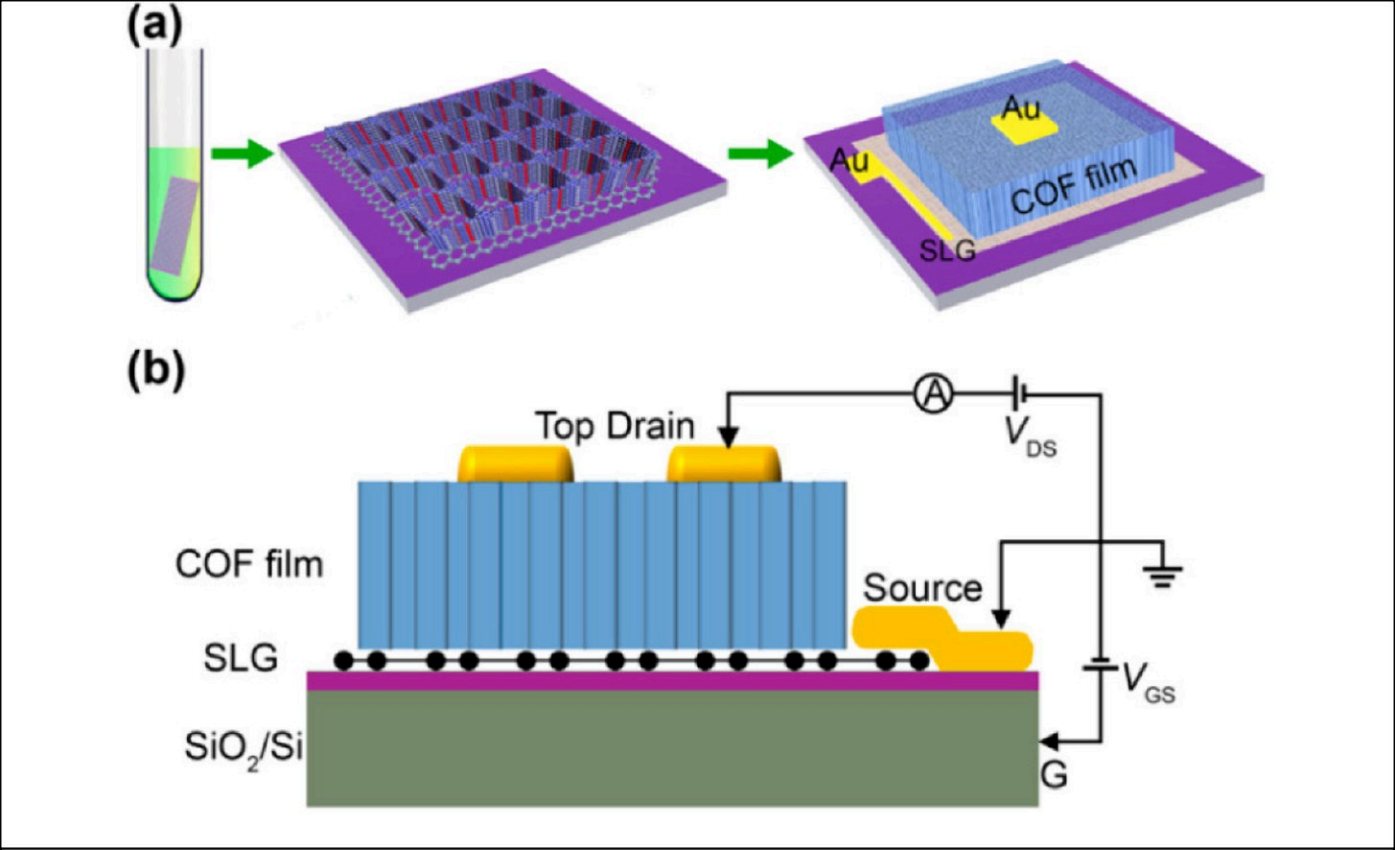
A COF-based vertical field effect transistor (VFET). (a) Schematic diagrams of synthesizing TFPy-PPDA COF film on SLG/SiO_2_–Si for VFET device fabrication. (b) Side view of a COF/SLG-VFET device. Reproduced from ref (305). Copyright 2017 American Chemical Society.

Finally, ambipolar characteristics in a vertical field effect transistor were found for a pyrene COF with imine linkage,^305^ showing that COFs can also withstand high current densities (Figure 11). The above exciting results (and those detailed in Table 2) clearly indicate that the field has entered a new era of high-mobility COFs. Among the COFs showing carrier mobilities in devices exceeding 1 cm^2^/(V s), those featuring pyrazine linkage motifs predominate. In this sense, presently pyrazine-linked COFs can thus be regarded as most established structures showing robust device mobilities above 1 cm^2^/(V s).

Some of the highest mobilities measured for COFs are comparable to those of high-mobility metalorganic frameworks (MOFs)^306,307^ or coordination polymers.^308^ A brief qualitative comparison with MOFs is therefore appropriate. In MOFs the π-conjugation is formally broken between the ligands due to the polar metal coordination bond. Still, metals can mediate electron tunneling through these bonds^309^ and finite in-plane band dispersion can occur in 2D MOFs similar to 2D COFs. In addition, the coupling of metal atoms across the layers of 2D MOFs potentially induces high mobilities and conductivities for doped systems with metallic charge transport characteristics.^306^ This coupling appears to be similar to the interplane coupling between the π-orbitals of some COFs.^301^ Also, the anisotropy of charge transport in 2D COFs and 2D MOFs is expected to share common features based on their predicted electronic structure.

### A Perspective to Study Transport at Dirac Cones

Besides the impressive developments discussed above and the substantial increase of the absolute mobility values of charge carriers in COFs, electronic transport at Dirac cones has not yet been systematically studied. This would be interesting with a view on the analogy to the DCs of graphene with its intriguing electronic transport properties mentioned above and the additional chemical tunability of DCs in COFs, which is not easily possible in graphene (and variations such as nanographenes). However, in many systems, the DCs can be buried in the band structure,^199^ and their energetic distance to the Fermi level then makes it difficult to access DCs electrically. One way out of this conundrum would be to realize systems where the intrinsic Fermi level crosses the DCs. An alternative could be doping of COFs. In the broadest sense, the term doping would include both optically induced, electrically injected or chemically provided charges, while a narrower understanding implies chemical doping by impurities (i.e., dopant species of n- or p-type) with the purpose to shift the Fermi level and/or to increase the carrier density.^328^ Typical doping species for organic materials in general include alkali atoms, small molecules such as SbCl_5_, HCl, or iodine,^314,329^ but also small heterocyclic molecular dopants like TCNQ or F_4_TCNQ.^210,330^ However, to date not all of these have been experimentally realized in COFs (see Table 2). Metal–organic species such as cobaltocene have also been successfully applied for 2D polymers with naphthalene diimide moieties serving as electron acceptor.^219,331^

### Simulations of Charge Transport

Simulations of charge transport have been initiated in recent years,^223,233,332,333^ and so far, these rely mostly on the band picture and semiclassical scattering approaches such as in the deformation potential theory,^334^ or assume a parametric scattering time for traveling band electrons. We note that carrier mobilities are among the most challenging quantities to simulate theoretically and hence, simplifying assumptions are currently invoked, such as the presence of delocalized Bloch states or simplified models of vibrations restricting to an acoustic low-frequency phonon branch in the long wavelength limit.^334^ Its effect on the electronic bands determines the scattering time that may be used within a Boltzmann transport equation framework in the relaxation time approximation.^335^ It is clear that deformation potential theory involves a set of severe approximations, and it would be desirable to include all phonons.^336^ With these assumptions about electronic states in the systems, about the scattering behavior and about transport time scales among others,^337^ it is difficult to assess if this approach can still give a reasonable order-of magnitude estimate for carrier mobilities.^223^ Improvements of theoretical models are desirable but are complicated, on the one hand due to the large unit cells in COFs, and due to the challenging coupling of the electron motion to the vibrations on the other hand.^338^

While insights into the band structure provide important gains in understanding, one should not forget that calculating band structures tacitly ***assumes*** that the band concept of infinitely extended states is valid. This might be the case if all the atoms in the COF are strictly fixed—an important assumption in present calculations done for zero temperature and in the absence of additional structural or energetic disorder. Even if the latter conditions can be realized, at room temperature the COF building blocks are in motion with substantial vibrational amplitudes (as in other organic semiconductors^339,340^) and electron-vibration coupling (EVC) is known to be important for molecular solids with comparable electronic properties (albeit without covalent bonds).^341−343^ EVC and the resulting polaronic effects (such as band narrowing, satellite structures in the density of states and phonon progression in UV–vis absorption and emission) that are induced by strong EVC should be optimally invoked in the description of such materials including charge transport.^344,345^ Electronic states in organic materials can be partly^346^ or transiently localized because of their coupling to the vibrations.^347^ In 2D COFs, characteristics beyond idealized band structures are largely unexplored, but EVC needs to be taken into account for an enhanced understanding of the derived physical properties ranging from charge transport to opto-electronic features.^348^

### Exciton Dynamics in COFs

Typically, excitons, i.e., excited electron–hole pairs (see above), are strongly bound in organic semiconductors due to the low dielectric constant of the organic materials (the “excitonic effect”), ultimately hindering exciton dissociation into free charge carriers available for photovoltaic or photocatalytic applications. A recent study aimed at controlling the behavior of excitons in tetraphenylpyrene/linear acceptor COFs (representing Lieb lattices) found that this is feasible by reducing the band gap through suitable D-A combinations in the COF, resulting in significant activity in photocatalytic water splitting,^349^ while also linker modifications can modify excitonic properties.^350,351^

The charge carrier dynamics of a few selected COFs have been studied theoretically^352^ and experimentally by means of transient absorption spectroscopy, as well as microwave conductivity and pump–probe terahertz spectroscopy, providing information about the charge carrier dynamics all the way into the ultrafast fs-time domain.^304,353−357^ An important factor here is the exciton binding energy, which can be strongly influenced by the structure of the COF. For example, phenothiazine-based COFs were shown to exhibit a very low effective binding energy of only 50 meV in temperature-dependent photoluminescence measurements, leading to efficient exciton dissociation and photocatalysis.^358^ COF-integrated donor–acceptor moieties in benzo-bisthiazole bridged COFs assist in exciton dissociation and the generation of longer-lived photoinduced charge carriers, which were employed in photocatalytic hydrogen generation.^359^ Similarly, a ketene-cyano D-A pair in a COF nanosheet leads to strongly prolonged charge carrier lifetimes and enhanced photocatalytic efficiency in photocatalytic hydrogen generation.^360^ Excitonic effects could also be tuned by varying the central metal in porphyrinic COFs.^361^ Here, Zn(II) favors singlet to triplet exciton conversion, while Ni(II) promotes exciton dissociation into hot carriers upon photoexcitation. Hence, these different COFs can engage in vastly different photocatalytic pathways in the photooxidation of terpinenes and other photooxidations.

## Optoelectronic Behavior

### Understanding and Controlling Photoluminescence

**Figure 12:**
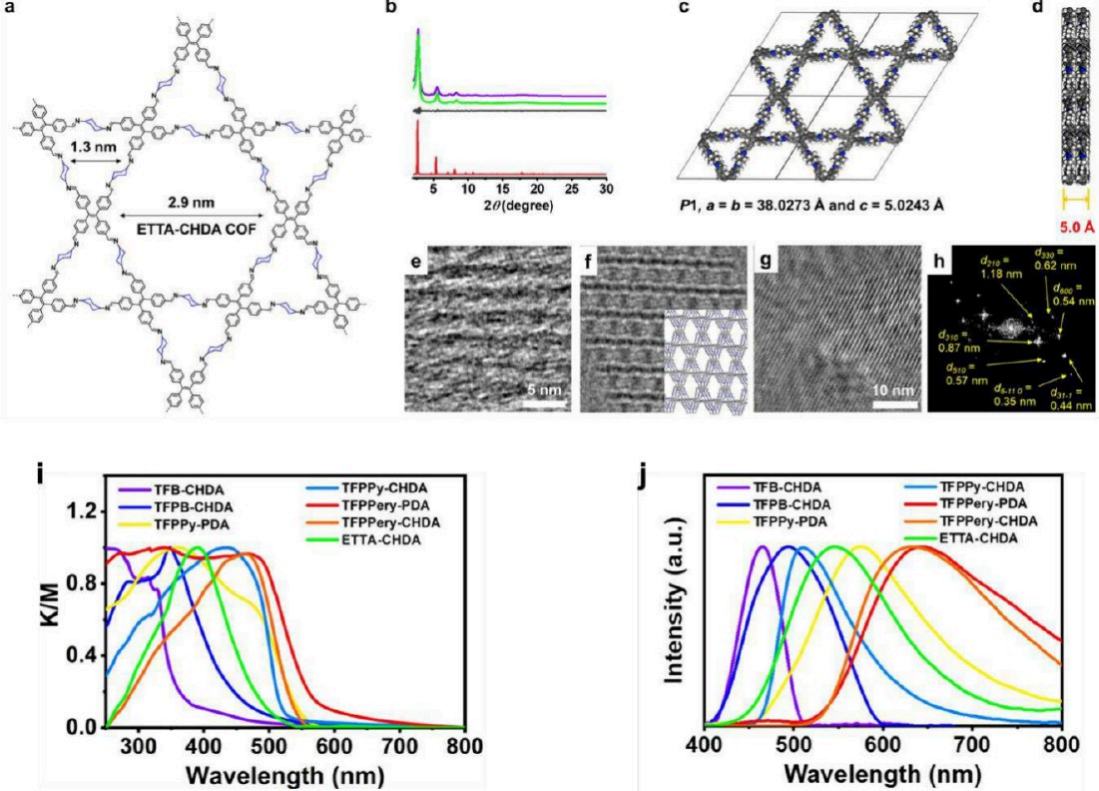
Integrating saturated trans-1,4-diaminocyclohexane (CHDA) linkers in 2D COFs with large interlayer distance and interrupted conjugation to boost luminescence. Top panel: (a) tetraphenylethylene (ETTA-CHDA) COF, (b) PXRD and eclipsed AA-simulated pattern, (c, d) optimized crystal structure, (e-h) TEM images (f: simulated) and FFT data of ETTA-CHDA. (i) UV–vis diffuse reflectance spectra, (j) luminescence spectra of different CHDA-linked COFs. Adapted from ref (367). CC BY 4.0.

The above-discussed fundamental physical properties of semiconducting (2D) COFs will also determine their behavior in photoluminescence (PL), which is strongly impacted by the stacking and aggregation motifs of the individual 2D layers.^362^ The stacking could, for example, result in either H- or J-type aggregation^363^ of the layers, depending on relative layer shifts and mutual layer orientation. Also, the luminescence is expected to be controlled by the relevance of nonradiative decay channels, which are often aided by vibrational or rotational modes in the lattice.^364^ The impact of vibrational modes on the relaxation of excited systems is also important for an understanding of the aggregation-induced emission (AIE) behavior that has been observed, for instance, with COF building blocks such as tetraphenyl ethylene (TPE or ETTA)^365^ or in 1D COFs with nonlinear edges and high-symmetry vertices,^366^ whose behavior is strongly influenced by their embedding or integration into the COF lattice. Moreover, it has been found that saturated (cyclohexane) linker nodes in 2D COFs can dramatically increase their PL quantum efficiency (Figure 12).^367^

A complementary approach toward achieving high fluorescence quantum yield^368^ is the combination of rigid fluorescent nodes with flexible nonplanar building blocks.^369^ The phonon spectrum of COFs can also be significantly modulated by substituting hydrogen with deuterium in the lattice, leading to strongly enhanced luminescence quantum yields.^370^ The resulting slower vibrations are believed to impede relevant nonradiative deactivation channels.

Not only the molecular COF structure but also the overall morphology can be key to achieving a certain optoelectronic behavior—for example by enabling the fast growth of large single crystals (up to 0.1 mm) in supercritical CO_2_. This morphology allows for the observation of polarized photoluminescence and polarized second-harmonic generation emanating from the single crystals of different 3D COFs, combining tetraphenyl methane with several other building blocks.^371^

Highly emissive COFs were also demonstrated by employing a module-patterned approach for assembling sp^2^-carbon linked hexagonal COFs with large, multitopic building blocks.^316^ A detailed mechanistic study shows that a suitable combination of planarity, sp^2^-carbon conjugation, dipole moment orientation and interlayer aggregation of chromophores control both the absorption, exciton dynamics and luminescence features of COFs.^372^ Favorable interlayer packing was also invoked to explain high luminescence quantum yields in an unusual stair-stepped COF integrating a Z-shaped building block.^373^

Beyond layer stacking, the internal pore surface can also be modulated to control luminescence. Modifying the pore walls to induce resonance, tautomerization or hyperconjugation effects resulted in the ability to emit three primary colors, and combining the perturbation of multiple sites resulted in diverse, tunable emission colors.^374^

Taking advantage of the modularity of the COF construction paradigm, the integration of axially chiral (linear) linkers with symmetric benzenetriacetonitrile nodes resulted in chiral COFs emitting circularly polarized luminescence.^375^ Notably, chirality could also be imparted into a COF by means of chiral amine inducers employed during COF nanosheet synthesis, which could also be postsynthetically modified with additional chromophores.^376^ These COFs also exhibited circularly polarized luminescence.

Numerous ***applications and functionalities*** of organic semiconductors are based on their optoelectronic features. Here we provide a brief overview of such functionalities for COFs, while the reader is directed to the original literature for more detailed discussions.

An intriguing property of COFs is the ability to recombine injected electrons and holes such that ***electrochemiluminescent*** behavior is achieved.^377,378^ The phenomenon of electrochemiluminescence (ECL) is a powerful transduction technique, depending critically on the generation of an excited emitter via charge transfer between electrochemical reaction intermediates of the emitter and a coreactant/emitter. These systems can provide sensitive analytical strategies for detecting and tracing biomarkers.

The photophysical properties of COFs can be controlled to achieve near-infrared light fluorescence imaging through two-photon induction by combining excitation states, for example with a benzothiadiazole-containing D-A COF,^379^***two-photon absorption*** (e.g., by integrating triarylamine nodes and p-phenylenediamine in a COF, leading to anisotropic serrated layer stacking and collective alignment of their transition dipoles),^380^ or octupolar three-branched node containing sp^2^-carbon linked COFs,^381^ and generally ***nonlinear optical behavior***,^382,383^ depending on the nature of the building blocks and the connectivity in the COF lattice. An intriguing combination of electronic properties and redox behavior of COFs can be observed in the phenomenon of ***electrochromism***. Here, COFs are electrochemically reduced or oxidized and upon reduction or oxidation of the lattice the optical spectrum can change dramatically and at high speed due to rapid ionic diffusion, being of interest in the design of displays and switchable windows.^175,384^

***Triplet photosensitizers*** are of growing interest with a view on controlling photochemical reactions. Such systems can be generated by embedding lone-pair D-A bonds into conjugated COFs, for example based on COFs with triazine nodes.^385^ Here, the nitrogen atoms in triazine could be directly coupled with oxygen atoms (via ammonium persulfate) to yield an inner molecule lone-pair donor–acceptor bond of triazine N-oxide, with oxygen centered radicals, enhancing singlet oxygen generation and photooxidation of sulfides. Control of spin behavior is also key in enhancing spin-forbidden phosphorescence in COFs.^386^ Using the concept of covalent doping via copolymerization of halogenated (heavy atom effect) and unfunctionalized building blocks in COF-1, highly phosphorescent COFs could be demonstrated, with efficient oxygen sensing capability.^387^ In addition, extremely long room-temperature phosphorescence has been shown in ref (388).

### Heterojunctions and Interfacial Electron Transfer

The construction principles of 2D COFs with their stacked columns of building blocks would appear to be attractive model systems for the construction of ***periodic bulk heterojunctions***. Due to the typically short exciton diffusion lengths, bulk heterojunctions are the enabling components in organic photovoltaic devices.^1^ In these devices, bulk heterojunctions are traditionally formed by the spinodal decomposition of donor and acceptor polymers or small molecules into nanoscale domains of donor and acceptor phases that allow diffusion of excitons toward domain boundaries such that charge separation can occur within their lifetime. First demonstrations of COF-based heterojunctions with light-induced charge separation have been reported,^174,389^ but the systems still require optimization of their optoelectronic features, including the recombination dynamics, to reach attractive photovoltaic efficiencies. Generally, donor–acceptor (D-A) type COFs are of interest in diverse fields including photocatalysis, photothermal therapy, and electronic devices.^390^ Heterojunctions of interest in photocatalysis will be discussed in the following section.

We note the vast scope of opportunities for the design of ***chemical sensors***(391) resulting from the optoelectronic features of COFs combined with the ability of their large pore systems to absorb molecules. For example, any suitable interaction of molecules diffusing into the pore space of COF thin films can serve as tranducing principle for say, ***optical sensors***,^392^ while chiral COFs can serve to detect circularly polarized light.^393^ Often, quenching of the COF’s PL by molecules diffusing into its pore systems is used for their detection, or, vice versa, PL turn-on phenomena upon adsorption are utilized.^394^ A different transduction principle is a change in the optical absorption or emission spectrum as a result of absorbing molecules in the COFs, based on the solvatochromic effect.^395^

## Photocatalysis and Photoelectrochemistry

Based on the structural and electronic features of COFs, one of their prominent applications is photocatalysis.^396^ The general principle is to use the COF as an antenna system which will absorb light of appropriate energy, create excitons, and separate these into electrons and holes at the interface to a catalytic center. Since COFs could be considered as an assembly of localized antenna systems with localized catalytic centers, which do not necessarily need to be electronically connected, they do not need to be highly conductive. In contrast, COF-based photoelectrochemistry requires conducting electrodes (see below).

At the center of photocatalytic research are two types of reactions. First, aiming at overall water splitting into O_2_ and the solar fuel H_2_ from earth-abundant H_2_O. However, since this is a highly complex process, much effort has been focused on the reducing half reaction of hydrogen evolution, which requires a sacrificial electron donor (SED) to resupply electrons. Second, the reduction of the greenhouse gas CO_2_ to CO or other higher-energy chemicals (or fuels). In this section, we present recent key results and latest trends toward future developments in COF-based photocatalysis and photoelectrochemistry.

As a first step toward developing a COF photocatalyst, tuning the bandgap and band positions toward the desired catalytic reaction and simultaneously optimizing (solar) light absorption is primarily done by the choice of molecular subunits and their linkage motif. Further fine-tuning has been achieved by extension of conjugated systems and introducing donor–acceptor (D-A) push–pull effects.^397^ As a cautionary note, the reader should be aware that comparing the photocatalytic performance between systems can be misleading due to differences in spectral irradiation, irradiation power, dilution of the suspended catalyst in solvent, stoichiometric ratio between catalyst and SED, type and reaction rates of the SED, stirring rates, and drastically different levels of light scattering and absorption depending on the suspended COF, geometry and volume of the reactor, and on the geometry of the light sources used for irradiation. A recent study illustrates the strong impact of particle size on photocatalytic efficiency.^398^

### Photocatalytic Hydrogen Generation

By linking electron-rich with electron-deficient subunits, ***D-A heterojunctions*** are established that significantly promote charge separation. Thereby, hydrogen evolution reaction (HER) rates of over 1 mmol g^–1^ h^–1^ from H_2_O without any metal cocatalyst could be achieved.^399^ Similarly, a fully conjugated 3D COF has been constructed with integrated D-A heterojunction from an electron-rich cyclooctatetrathiophene-derivative and benzidine, which demonstrated metal-free HER activity of 40.4 mmol g^–1^ h^–1^.^168c^ Considering linkage chemistry, a 4-fold activity improvement was achieved by postsynthetically transforming the N-acylhydrazone linkage between terephthalohydrazide-derivative and 1,3,5-triformylbenzene (TFB) subunits to the stable and conjugated oxadiazole linkage.^75^ Furthermore, several groups have established that cyano-substitution on fully conjugated alkene linkages^33,400^ and on β-ketoenamine linkages^401^ can improve the charge separation, reduce recombination and promote the HER activity. For example, a fully C=C conjugated D-A COF made from 1,3,5-tris(4-formylphenyl) triazine (TFPT) and 1,4-phenylenediacetonitrile (PDAN) demonstrated HER activity of 107 mmol g^–1^ h^–1^ from H_2_O when used with Pt NP loading of 12 wt %.^402^ Similarly, introducing an increasing number of β-ketoenamine linkages could improve the planarity, tune the band gap, reduce recombination and thus improve the activity.^129^ Linking TAPB with 2,4,6-triformyl resorcinol, thus only enabling two irreversible enol-to-keto tautomerizations per node, resulted in highly crystalline COF formation through intermediate macrocycles and HER activity of 19.8 mmol g^–1^ h^–1^ from H_2_O with only 0.1 wt % Pt.^403^ By bridging electron-rich benzo[1,2-*b*:3,4-*b*′:5,6-*b*″]trithiophene-2,5,8-tricarbaldehyde and electron-deficient TFPT with PDAN, a single fully conjugated three-component COF was achieved, with HER activity of 70.8 mmol g^–1^ h^–1^ with 1 wt % of Pt.^404^ Furthermore, it was demonstrated for imine linkages that constitutional isomerism of the linkage itself in D-A COFs can have a dramatic (10-fold) impact on the photocatalytic performance through shifts in the conduction band minimum, which can likely be extended to other asymmetric linkage motifs.^405^

**Figure 13:**
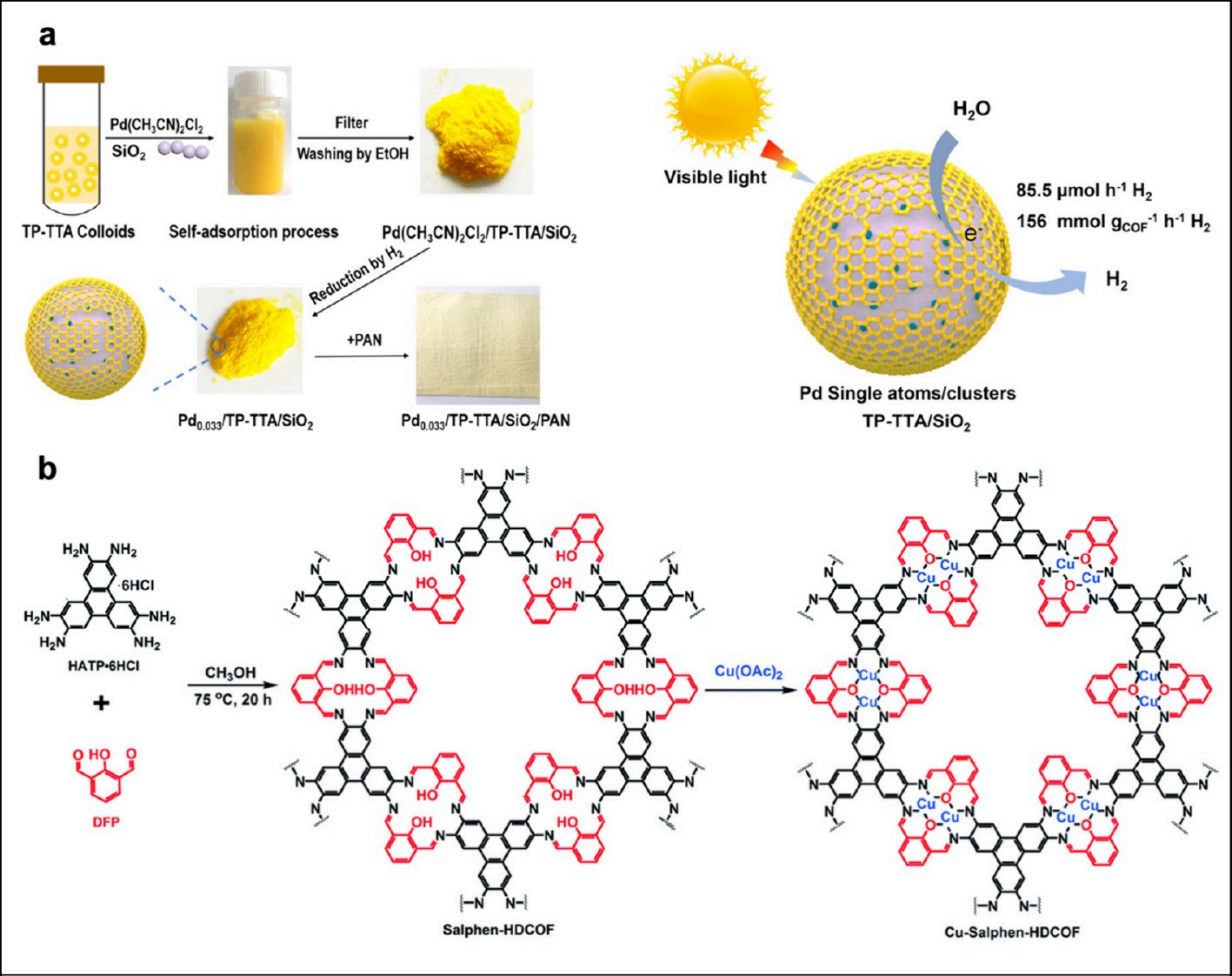
(a) Scheme of Pd_0.033_/TP-TTA/SiO_2_ synthesis and photocatalysis and (b) the synthesis and loading of a double-vacancy COF. Adapted from (a) ref (408) and (b) with permission from ref (411). Copyright 2022 American Chemical Society and 2020 Royal Society of Chemistry, respectively.

Despite the possibility to avoid any cocatalyst and still provide HER activity, most photocatalytic COFs depend on ***cocatalysts*** to reach their best potential or to enhance the selectivity toward specific reactions (as indicated by the Pt NPs employed in the above examples). One method to introduce cocatalysts into a COF is the undirected adsorption into the pores, which can be favorable if steady replenishment of fresh cocatalyst is desired.^406^ Furthermore, in situ photodeposition of Pt clusters was established by introducing adsorption sites that allowed for reduction of Pt^2+^ through photogenerated electrons, thereby exposing a large active surface of Pt and facilitating electron transfer, resulting in an HER activity of 42.4 mmol g^–1^ h^–1^ with 1 wt % Pt.^407^ To further optimize metal cocatalyst usage, Pd single atoms or clusters were formed on layers of a COF made from TFPG and 2,4,6-tris(4-aminophenyl)-1,3,5-triazine (TAPT). The layers were (self-)adsorbed on a SiO_2_ sphere together with a Pd^2+^ precursor, which was then reduced to Pd, resulting in an HER activity of 156 mmol g^–1^ h^–1^ and apparent quantum efficiency (AQE) of 7.3% at only 0.033 wt % Pd loading (Figure 13a).^408^

An alternative approach is the integration of specific docking sites for cocatalysts. By postsynthetic pore wall functionalization and subsequent covalent binding of cobaloxime-complexes, tight interaction between COF and cocatalyst was enabled, which enhanced the activity and limited degradation, resulting in improved HER activity and stability compared to physisorbed cocatalyst.^409^ Implementing single docking sites for metal complexes, bipyridine (bpy) was integrated into an sp^2^-carbon linked COF with TFB to stabilize Pd-NPs, whereby a high HER activity of 98.2 mmol g^–1^ h^–1^ was demonstrated.^410^ Furthermore, using additional intercalated Ni at the imine linkage of a COF incorporating the donor pyrene and the acceptor fluorenone, the polarization electric field which drives exciton dissociation could be increased 3-fold in addition to the D-A effect in the COF. This resulted in increased exciton dissociation and free charge carrier transfer to the active sites. With 3 wt % Pt NPs, a remarkable 198 mmol g^–1^ h^–1^ HER rate and 43.2% AQE at 420 nm was demonstrated.^320^ Considering access to catalytic sites, a high HER activity was demonstrated by designing a COF from trigonal hexaaminotriphenylene and 2,6-diformylphenol, wherein every linkage site contained two separate Cu docking sites for metalation, thus doubling the number of active sites per linkage (Figure 13b). Through additional delamination (exfoliation) into nanosheets to increase access to the active sites and to reduce charge transport distances, and without any noble-metal cocatalyst but with fluorescein as photosensitizer, an HER rate of 37 mmol g^–1^ h^–1^ was achieved.^411^

Moreover, by implementing the zwitterionic moiety *N*-sulfopropyl-2,4,6-trimethylpyridinium into the backbone together with TFPT, a hydrophilic sp^2^-carbon linked COF was constructed that showed record high HER of 288.8 mmol g^–1^ h^–1^ and 47.1% AQE at 420 nm with 1 wt % Pt as cocatalyst and ascorbic acid as sacrificial agent in aqueous solution.^412^

A very different approach was demonstrated by tackling the rate-limiting step of electron transfer from the COF to the cocatalyst.^413^ A β-ketoenamine linked COF from diamino-2,2′-bipyridine and TFPG was synthesized and subsequently some of the bipyridine units were quaternized into cyclic diquats, known electron transfer mediators, implying that they can efficiently accept electrons from the COF framework and transfer them to a Pt cocatalyst. Hereby, the COF also isolates the diquats in the framework, thereby preventing detrimental dimerization. An HER rate of 34.6 mmol g^–1^ h^–1^ was achieved.

### Photocatalytic CO_2_ Reduction

**Figure 14:**
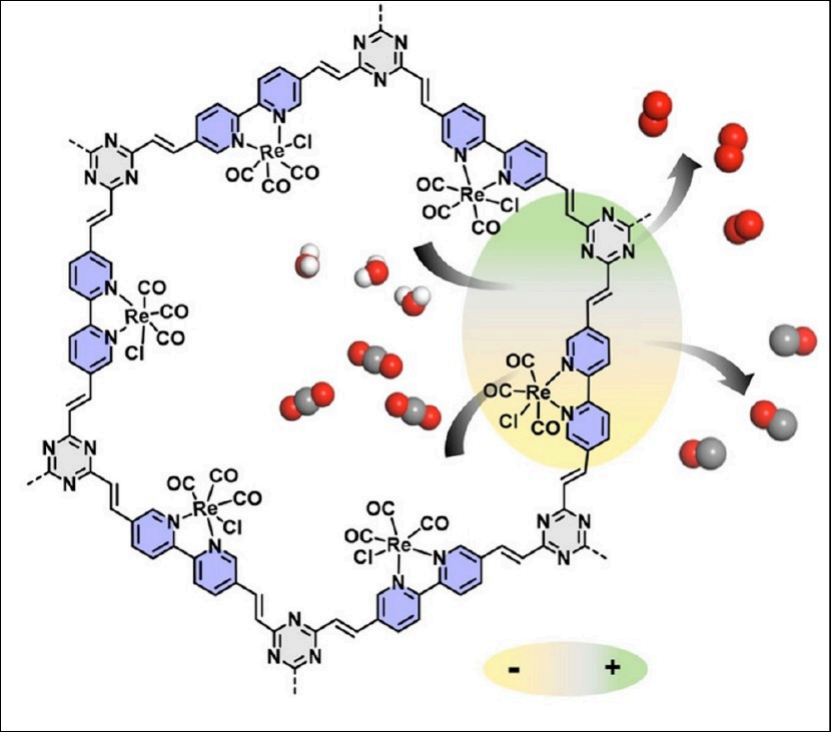
Illustration of the mechanism of photocatalytic CO_2_ reduction by using a Re-loaded D-A COF as photocatalyst. Reproduced with permission from ref (417). Copyright 2023 Wiley-VCH.

COF design principles similar to those above have been applied toward achieving CO_2_ reduction.^414^ However, in addition to activity, here the selectivity of the reaction is very important since the reduction can lead to multiple products and unwanted byproducts. For example, by improving the intrinsic π-conjugation via introduction of ethynyl-bridges and vinylene linkage in the COF, a metal-free CO production rate of almost 0.4 mmol g^–1^ h^–1^ was realized from CO_2_ and H_2_O without sacrificial agents.^415^ Introducing docking sites into the framework, a COF including a bpy subunit was synthesized which was able to coordinate a rhenium-containing moiety and outperformed the corresponding molecular photocatalyst [Re(bpy)(CO)_3_Cl].^416^ By linking the same Re-loaded bpy subunit to the strong acceptor moiety triazine, the internal charge separation was facilitated, resulting in a CO production rate of almost 0.2 mmol g^–1^ h^–1^, unprecedented from H_2_O, with 100% selectivity and a simultaneous O_2_ production rate of 0.09 mmol g^–1^ h^–1^ (Figure 14).^417^

**Figure 15:**
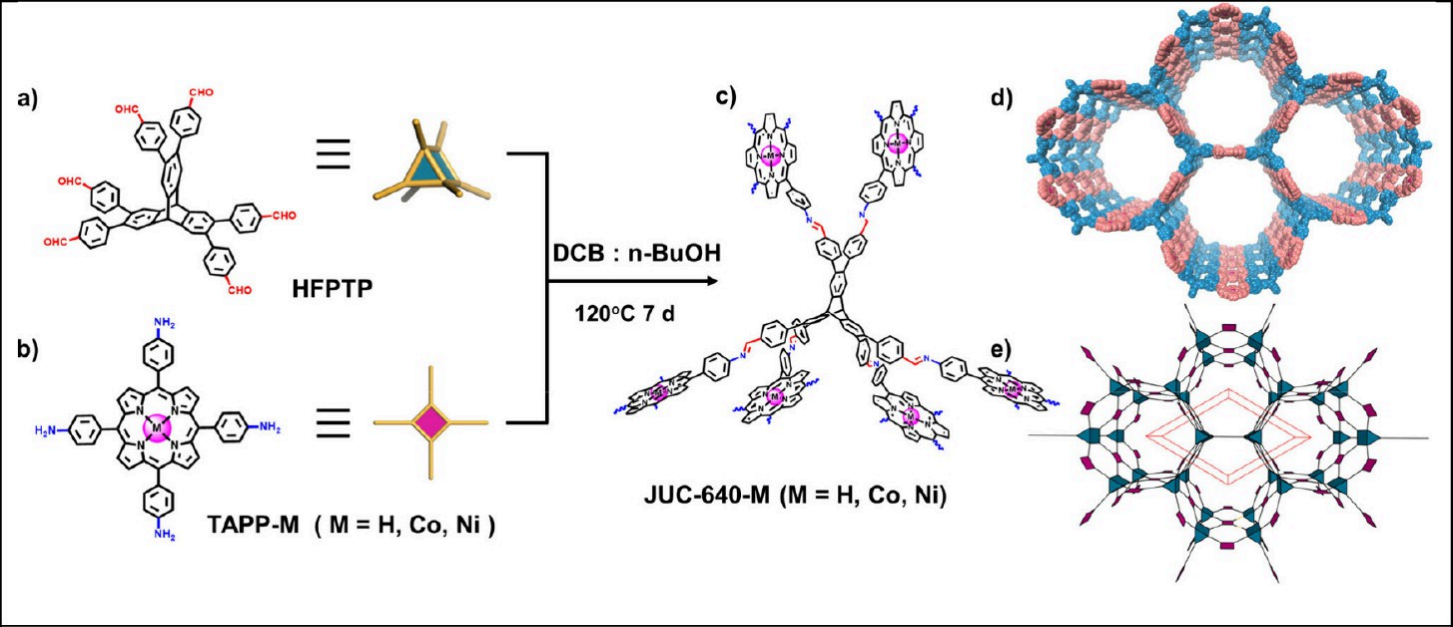
Synthesis scheme of the 3D porphyrin COF JUC-640-M. Reproduced from ref (420). Copyright 2023 American Chemical Society.

Phenanthroline was integrated into a COF-5 backbone to synthesize bifunctional La–Ni sites through postsynthetic adsorption. Directional charge transfer between these double-atomic sites increased CO production by over 15 times with 98% selectivity.^418^ A Co^2+^-complexing COF made from 1,4-phenylenediamine (PDA) and 3,3‘,5,5′-tetraformyl-4,4′-biphenyldiol was synthesized, which was then chelated with a salicylideneaniline (SA) ligand.^419^ This COF-Co-SA system outperformed both the corresponding molecular catalyst and COF-Co, signifying the importance of the active site coordination environment. Cocatalyst-loaded porphyrin subunits have also been implemented, e.g., the large-pore 3D COF made from 2,3,6,7,14,15-hexa(4′-formylphenyl)triptycene (HFTPT) and Co^2+^-loaded 5,10,15,20-tetrakis(4-aminophenyl)porphyrin (TAPP) demonstrated a record-high CO production rate of 15.1 mmol g^–1^ h^–1^ with 94.4% selectivity (Figure 15).^420^ Moreover, a nanosheet-based approach was applied resulting in an increased CO generation rate of 10.2 mmol g^–1^ h^–1^ and 78% selectivity in aqueous media with an imine COF synthesized from Co-TAPP and 4,4-biphenyldialdehyde.^421^ First steps have also been taken toward the more complex multi-electron reductions of CO_2_ to methanol, formic acid, and methane, for example employing nitrogen-rich triazine, porphyrin and azine COFs, but generally the production rates and selectivities are still low.^422^ As already indicated above, porphyrin-based COFs have gained increasing attention as these macrocycles can chelate a multitude of single metal ions, thus, at least conceptually, creating single site catalysts and acting as efficient electron-transfer carriers (depending on molecular access due to stacking), for example for the photocatalytic reduction of CO_2_ to formate.^423^ An sp^2^-carbon conjugated metal-covalent organic framework constructed from the electron-deficient 3,5-dicyano-2,4,6-trimethylpyridine and the electron-rich metal cluster copper pyrazolate-4-carboxaldehyde demonstrated a relatively high CO_2_ to formic acid conversion rate of 0.3 mmol g^–1^ h^–1^ and 97.1% selectivity.^424^

### Additional Reactions

**Figure 16:**
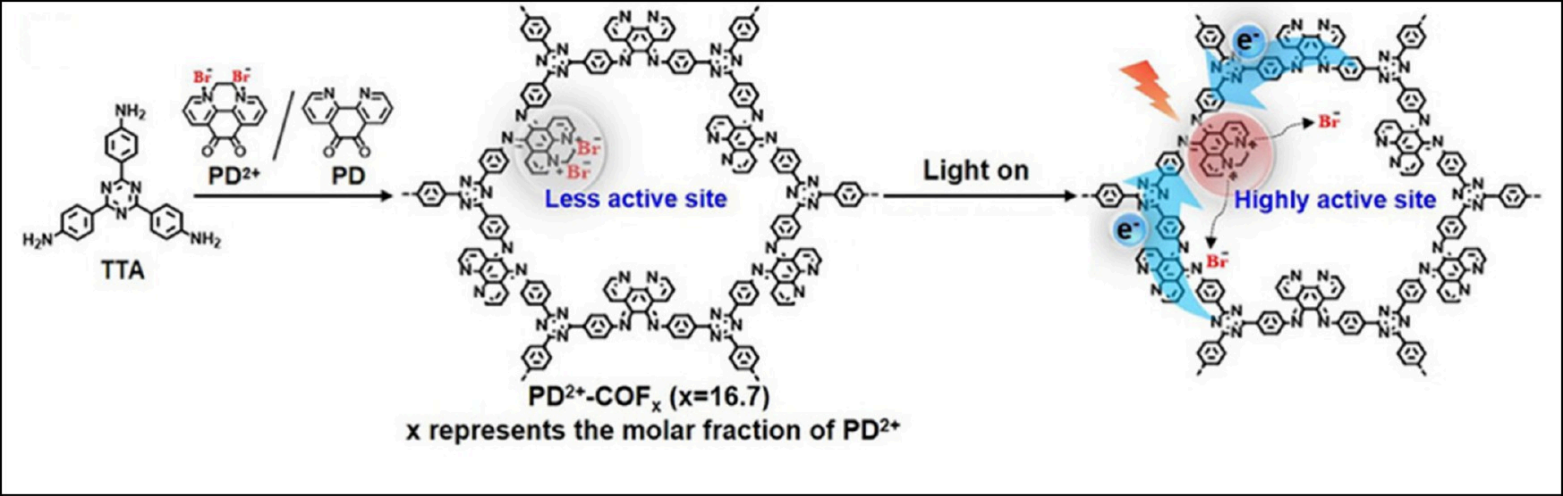
Schematic representation of photoinduced transformation of PD^2+^-COF_*x*_ into nitrogen cation motifs by bromide dissociation. Adapted with permission from ref (434). Copyright 2023 Wiley-VCH.

Further achievements in COF-based photocatalysis include C–C cross-coupling,^425^ C–N cross coupling,^426^ radical polymerization,^427^ aryl etherification,^428^ aerobic oxidation of sulfides,^429^ alcohol oxidation,^430^ uranium extraction,^117a,431^ and oxidative amine coupling.^432^ Typical design strategies again include pure COF-based organocatalysts enhanced through D-A heterojunctions or backbone engineering, site-specific binding of single or dual molecular (metal)catalysts, and attaching metal nanoparticles to the COF walls. Significant efforts have been directed toward improving photocatalytic H_2_O_2_ production,^433^ resulting, for example, in a rate of 12.0 mmol g^–1^ h^–1^ with a COF made from TTA and phenanthroline-5,6-dione quaternized with dibromo alkanes. Here, bromide ions dissociate upon irradiation, leaving the nitrogen cation sites which thus become more acidic, and thereby facilitating charge separation and transfer and favoring O_2_ adsorption (Figure 16).^434^

Similarly, high photocatalytic production rates have been achieved by either introducing robust quinoline linkages, thereby making the COF more stable and photocatalytically active under strongly oxidative conditions, or by optimizing the charge separation efficiency in D-A COFs via further modulation of the acceptor moiety with electron-withdrawing cyanide side groups.^435,436^

Exploring the realm of chiral synthesis, interpenetrated 3D COFs from either nitrilotris([1,1′-biphenyl]4-carbaldehyde) and tetra-*p*-aminophenylethylene (ETTA) or ethenetetrayltetrakis([1,1′-biphenyl]-4-carbaldehyde) and tris(4-formylphenyl)amine were synthesized. With an additional chiral MacMillan catalyst adduct the enantioselective α-alkylation of aldehydes could be efficiently promoted, an early example of asymmetric reactions.^437^ A chiral COF photocatalyst for asymmetric relay reactions was synthesized by combining Au–N-heterocyclic-carbene and chiral secondary amine subunits.^438^ Similarly, chiral COF “nanozymes” were constructed with postsynthetic covalent integration of either l- or d-histidine subunits.^439^ Depending on the histidine chirality, the oxidation of dopa showed strong selectivity for l- or d-dopa and for additional enantioselective reactions. This appears to be the first example of a chiral nanoenzyme with higher enzymatic activity than the corresponding natural enzyme.

### COF Hybrid Systems

Another class of intriguing COF photocatalysts emerges from the hybridization with different materials, making use of their distinct structural or functional advantages. Specifically, the hybridization of COFs with metalorganic frameworks (MOFs) has recently been explored, including either core–shell^440^ or covalently linked^441^ heteroframeworks for multiple photocatalytic reactions, whereby the latter heterojunction shows 23.4 mmol g^–1^ h^–1^ HER activity. Therein, the COF functions as photosensitizer and oxidizes the SED. Following the energy level alignment, the excited electron is transferred to the lower lying LUMO of the MOF and from there to protons for H_2_ evolution. In this so-called ***S-scheme***, the COF-MOF heterojunction itself strongly enhances the charge separation and thus facilitates photocatalysis. Furthermore, a heterojunction of a COF with lead-free halide perovskite-like (CH_3_NH_3_)_3_Bi_2_Br_9_ was demonstrated, in which the COF made from TAPB and 2,5-dimethoxy TA (DMTA) encapsulated and stabilized the perovskites, and the formed heterojunction facilitated charge separation and transport resulting in efficient photocatalytic polymerization of acryl-PEG.^442^ Molecular hybridization of COFs with other carbon-based photocatalyst moieties has been demonstrated using heptazine subunits^443^ (the *g*-C_3_N_4_ building blocks) and nanographene subunits (these constructs could also be viewed as D-A COFs).^444^ Generally, the enhanced surface exposure of these low bandgap moieties in the COF backbone together with the efficient charge transport resulted in notable photocatalytic activities. Moreover, passivation of *g*-C_3_N_4_ nanosheets by covalently linking the terminal free amino groups to COF layers resulted in strong electronic coupling and 46.4 mmol g^–1^ h^–1^ HER activity and 31.8% AQE at 425 nm with Pt NPs.^445^ Electronic hybridization has been achieved by creating van der Waals heterojunctions of 2D COF layers on nanosheets of *g*-C_3_N_4_, which allowed for efficient metal-free CO_2_ reduction since the hybridization reduced the bandgap energy while the heterojunction limited charge carrier recombination.^446^

**Figure 17:**
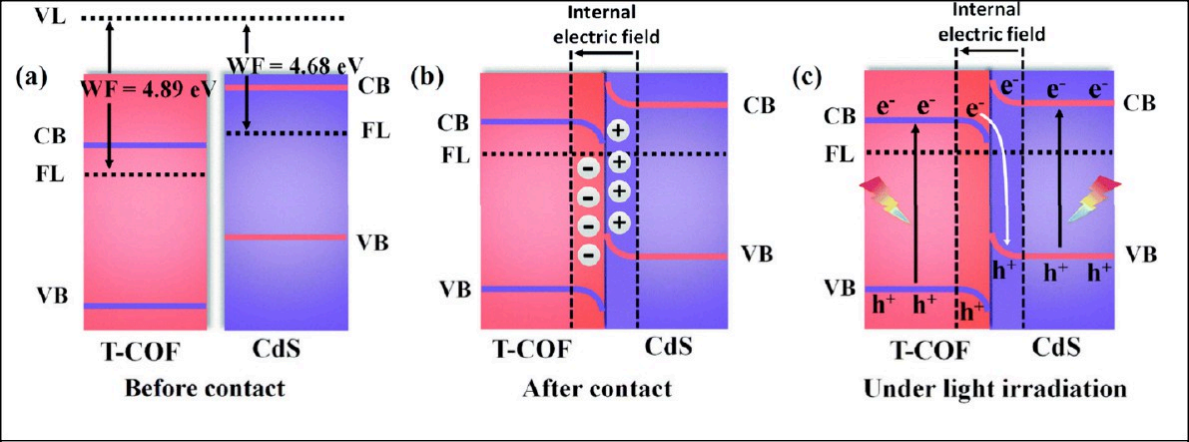
Schematic illustration of a Z-scheme heterojunction from a COF and CdS depicting the relative band positions and charge transfer process (a) before contact, (b) after contact, and (c) under light irradiation. Reproduced from ref (450). CC BY-NC 3.0.

Another strategy has been the design of ***Z-schemes***, in which the energy level alignment allows for the charge transfer from the donor’s LUMO to the acceptor’s HOMO, from where it can then be excited into an even higher LUMO. Thus, two subsequent excitation steps take place as in natural photosynthesis, thereby fully exploiting the semiconducting properties of COFs and other organic or inorganic materials while simultaneously facilitating spatial charge separation and catalytic reactions (Figure 17). Such a heterojunction was demonstrated by modifying a COF made from TAPB and 2,5-divinylterephthaldehyde with thiol groups, which then acted as nucleation sites for Au clusters and, via a dual excitation process, led to the efficient photocatalytic degradation of Rhodamine B and bisphenol A, which Au alone cannot achieve.^447^ Moreover, inorganic/COF heterojunctions with α-Fe_2_O_3,_^448^ WO_3_^449^ and CdS have been applied for efficient H_2_ evolution, the latter achieving a high AQE of 37.8% at 365 nm for water splitting and long-term stability since the core–shell structure protected the CdS.^450^ Remarkably, a Z-scheme heterojunction of a COF from TFPG and 3,7-diaminodibenzo[*b*,*d*]thiophene 5,5-dioxide chemically bonded to O-vacancy WO_3_ nanosheets demonstrated an HER activity of 593 mmolg^–1^h^–1^ and an AQE at 420 nm of 56.1%, which could be further implemented for overall water splitting reactions.^451^

Furthermore, applying water-oxidizing inorganic semiconductors such as TiO_2_, Bi_2_WO_6_, and α-Fe_2_O_3_ together with CO_2_-reducing COFs resulted in covalently bonded artificial synthesis systems reaching CO production rates of up to 69.7 μmol g^–1^ h^–1^ for a polyarylether COF made from 2,3,6,7,10,11-hexahydroxytriphenylene and 2,3,5,6-tetrafluoro-4-pyridinecarbonitrile.^452^ In time, such hybrid Z-scheme systems may have the potential to outperform both individual COF-cocatalyst systems and inorganic photocatalysts.

### Bioapplications

Demonstrating the diversity of COF-based photocatalysis, it was established that an acridine-based COF showed antibacterial photocatalytic properties in fish through generation of reactive oxygen species (ROS).^453^ Furthermore, multiple COFs have been implemented in photodynamic therapy concepts, wherein ROS are generated or released locally through photocatalysis to induce cancer cell apoptosis. Usually D-A COF NPs are designed, which could be further improved by exfoliation and tumor-targeting functionalization.^454^ Other examples include prior ^1^O_2_ generation and storage within the COF to be released at the tumor cells,^455^ luminol encapsulation to create a self-contained light source for ROS generation at cancer cells,^456^ and a combined approach where the dithioketal-linked COF’s ROS generation also caused self-dissociation and thereby chemotherapeutic drug release.^457^

### Photoelectrochemistry

A modification of the above photocatalytic paradigm is the construction of ***photoelectrochemical cells*** (PEC) where the oxidation and reduction processes are separated into different electrochemical compartments and the processes are localized at the electrodes, namely a photoanode and a photocathode. The anode, the cathode or both are coated with (typically COF-based) light absorbers, and the photoelectrochemical reactions can occur on either or both electrodes. Unlike in photocatalysis, this requires some electrical conductivity of the COF. One advantage of this concept is the ability to separate the products into two different compartments, or half-cells, which could be highly beneficial when splitting water. Crystalline COF nanofilms from TAPB and DMTA as photocathode for solar hydrogen production with the stack layout CuSCN/P3HT/COF/SnO_2_/Pt demonstrated an onset voltage of 1.06 V_RHE_ and a photocurrent density (*J*_Ph_) of 17 μA cm^–2^ at 0.7 V_RHE_ under 1 sun illumination, which is the current benchmark for COF photocathodes.^133b^ An electrophoretically deposited COF film from ETTA and benzo[1,2-b:4,5-b′]dithiophene-2,6-dicarboxaldehyde decorated with Pt nanoparticles exhibited a *J*_Ph_ of 128.9 μA cm^–2^ at 0.1 V_RHE_ with an onset voltage of 0.45 V_RHE_ under 1 sun illumination when implemented as photocathode for H_2_ evolution from H_2_O,^182^ based on prior work where the COF film was generated solvothermally.^458^ A photoanode was established by coupling a COF made from TAPT and TAPG to nanotemplated ZnO, whereby the heterojunction enhanced the *J*_Ph_ to 0.62 mA cm^–2^ at 0.2 V_RHE_ in photoelectrochemical water oxidation.^459^ Interestingly, a dioxaborole COF condensed from 2,3,6,7,10,11-hexahydroxytriphenylene and 2,1,3-benzothiadiazole-4,7-diboronic acid demonstrated remarkable performance in the PEC nitrogen reduction reaction with notable ammonia yield of 142.9 μg h^–1^ mg^–1^ at −0.6 V_RHE_ and a Faradaic efficiency of 91.6% at −0.4 V_RHE_ through establishment of the active boron site between donor and acceptor moieties.^460^

**Figure 18:**
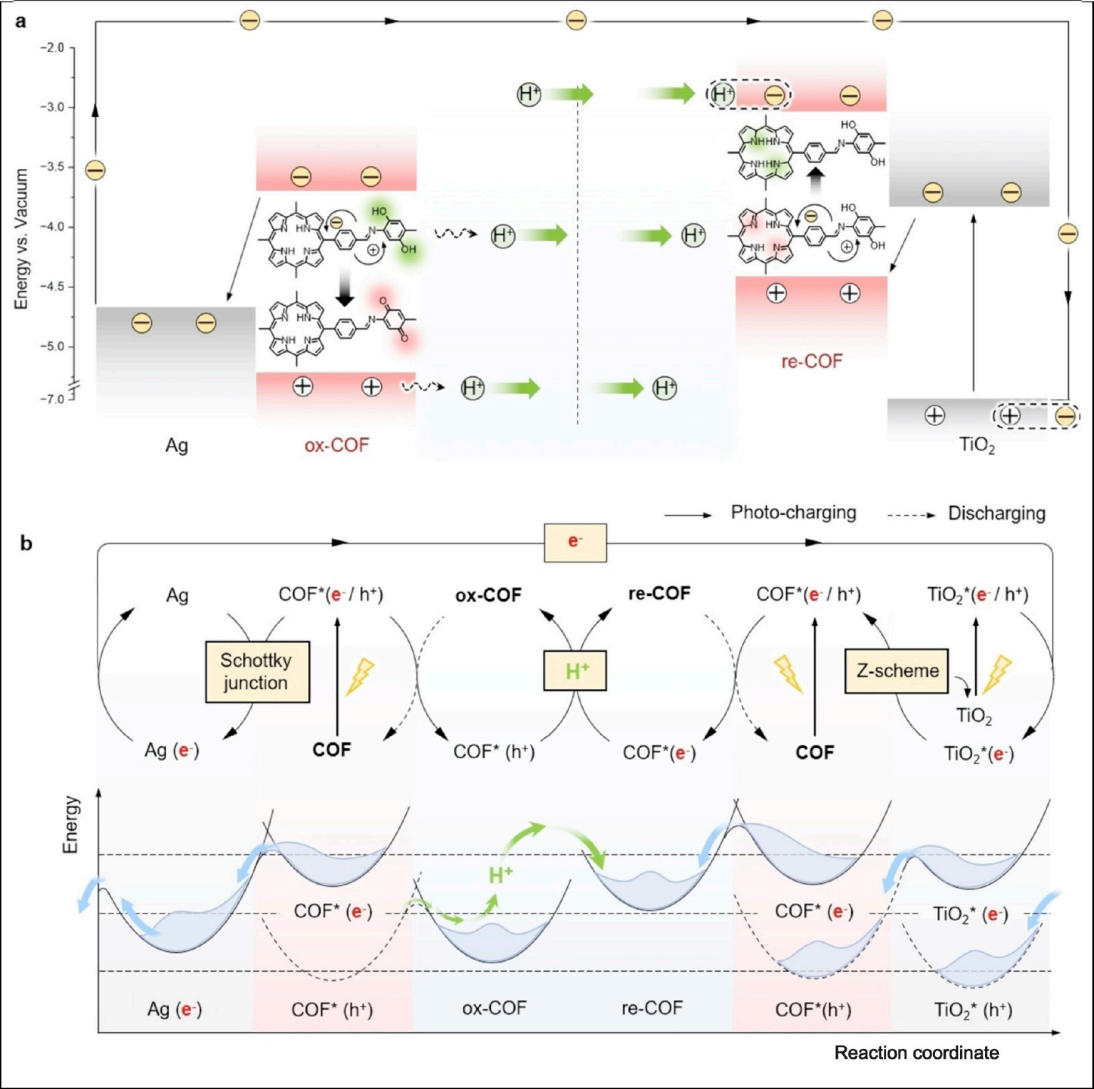
(a) Schematic illustration of the charge/discharge mechanism in a coupled solar battery and (b) the corresponding reaction steps and energy levels. Reproduced with permission from ref (462). Copyright 2023 Wiley-VCH.

Lastly, several COFs have now been successfully implemented in ***solar responsive batteries*** (allowing to electrochemically store solar energy absorbed and converted into free charges). Herein, a COF is used as part of the photocathode, e.g., a COF made from 1,4,5,8-naphthalenetetracarboxylic dianhydride and tris(4-aminophenyl)amine in a solar Li-ion battery.^461^ Moreover, a dual-redox active COF with efficient intramolecular charge separation made from n-type DHTA and p-type TAPP was coupled to both an Ag cathode and a KTiO_2_ anode, forming heterojunctions (Figure 18). Under 1 sun illumination, this resulted in a remarkable solar-to-electrochemical energy storage efficiency of 6.9%.^462^

## Conclusions and Outlook

Given the enormous body of work on diverse ordered microporous and mesoporous materials, the more “classical” features of COFs related to their pore systems, i.e., adsorption and diffusion of guest molecules and thermal organocatalysis, could be considered to be rather well understood. In contrast, a detailed understanding of their interaction with light and the ensuing quantum chemical processes is still challenging and requires substantial progress, as discussed throughout this Perspective and sketched in the following.

***Layer and sublattice stacking***. In 2D COFs, it would be highly beneficial to achieve even better structural characterization by means of single crystal analysis. The recent study on COF crystal growth in micelles^142^ promises progress in this area, hence allowing for a better understanding of the detailed and subtle impact of building blocks and linkage chemistry on layer packing distance and layer-to-layer correlation, twist angles, π–π interactions, defect densities, and their impact on optoelectronics. In 3D COFs, precise structural information on the dispersive molecular interactions in interpenetrated networks is of similar great relevance. In general, point defects such as missing linkers or intentionally changed local sites such as sparse binding of metal ions to coordinating sites, “doping” with isometric but different linkers, or sparse ionic sites introduced by postsynthetic modification will all have widely varying effects on optoelectronic properties, depending on both the nature of the COF and of the point defect. Focusing on grain boundaries, microscopic insight into their impact on carrier transport is currently missing. Generally speaking, these extended defects should be decisive for macroscopic carrier transport that is measured across grains. They can lead to a drop in the conductivity by several orders of magnitude because, typically, grain boundaries cannot be avoided by carriers along their transport pathways, resulting in trapping.

On the other hand, grain boundaries may be less important for optoelectronic properties such as linear optical absorption, which are expected to be averaged over crystalline and disordered sample domains. That is, if the structure is dominated by crystalline domains the boundaries will be mainly visible in the optical properties by a broadening effect even at small grain sizes. For this reason, it is highly desirable to characterize defects in COFs in general, which would lead to a better microscopic understanding of carrier transport and related properties.

***Controlling conjugation*** and understanding its impact on ***band gaps*** and ***charge carrier mobility*** in 2D and 3D COFs is still an emerging theme, requiring excellent structural and physical characterization as well as theoretical modeling. In 2D COFs, this regards in-plane vs out-of-plane mobility, while in the emerging group of 3D conjugated COFs, this regards along-bond vs dispersive interactions in interpenetrated systems and their impact on the band structure.

***Growing COF oriented thin films*** is of great importance for future device integration but still an art; a better understanding of wetting energies of building blocks and/or nuclei on diverse substrates and their impact on growth is highly desirable, as well as control over domain size and nature of grain boundaries. In some cases, using symmetry relations, e.g., with graphene,^179a^ or even heteroepitaxy/topology templating on MOF or COF crystals^463^ could offer exciting opportunities.

Theoretical challenges are still manifold due to the complex molecular framework systems, while COF synthesis and their characterization are being pursued at high pace. Simulations of electronic properties need to overcome the band gap problem of DFT with more elaborate methods. Excitons and their binding energy are even more challenging to simulate because of the Coulomb interaction and two-particle nature of the description. Finally, simulation approaches of charge carrier transport need to be more efficient and accurate. These can be regarded prerequisites for investigating the influence of topologies or doping on COF properties,^464^ while, at the same time, screening approaches should be further developed.^465^ All this is required to provide complementary theoretical insights and full understanding of electronic, optical and transport properties of COFs, ideally supported by precise measurements on well-defined model systems.

All of the above-mentioned steps will be of key importance toward the optimized design and applications of COFs in photon-induced processes such as photocatalysis and photoelectrocatalysis. While the design of D-A COFs offers a highly promising strategy for achieving efficient charge carrier separation, it is still difficult to precisely predict the relevant properties of COFs from their molecular building blocks and framework structure, especially when considering the stacking of 2D layers or of sublattices in 3D COFs. However, this will be necessary to fully exploit COFs regarding (metal free) photocatalysis with high specificity but also in more complex designs including the use of hybrid heterojunctions, with the COF often employed as photoabsorber and electron donor. Nonetheless, the promise of COFs with their intriguing interplay of structural, optoelectronic and morphological features has already been demonstrated in a wide range of processes ranging from efficient H_2_ evolution to asymmetric photocatalysis in synthetic chemistry.

Finally, improved understanding and control of the above structural and optoelectronic features is expected to enable access to a wide range of functionalities and devices, as discussed in the prior sections of this Perspective. To select one challenging theme, building periodic bulk heterojunctions for photovoltaic or COF-LED devices requires control over a whole set of optoelectronic features, including building blocks and linkage chemistry leading to a suitable band gap (light harvesting), oriented growth for achieving aligned stacks of donors and acceptors separated by suitable tunnel barriers, favorable low recombination losses (for photovoltaics), and high charge carrier mobilities. Employing and further developing a suitable selection of the strategies and tools discussed in this Perspective, these goals should be within reach. This example also illustrates the great promise of the deterministic assembly of COF-based crystalline organic semiconductors for optoelectronic applications.
